# Visual Feedback of Object Motion Direction Influences the Timing of Grip Force Modulation During Object Manipulation

**DOI:** 10.3389/fnhum.2020.00198

**Published:** 2020-05-29

**Authors:** Simone Toma, Veronica Caputo, Marco Santello

**Affiliations:** ^1^School of Biological and Health Systems Engineering, Arizona State University, Tempe, AZ, United States; ^2^Department of Biomedical and Neuromotor Science, University of Bologna, Bologna, Italy

**Keywords:** digit forces, object manipulation, sensory integration, visual gravity, predictive grip force control

## Abstract

During manipulation, object slipping is prevented by modulating the grip force (GF) in synchrony with motion-related inertial forces, i.e., load force (LF). However, due to conduction delays of the sensory system, GF must be modulated in advance based on predictions of LF changes. It has been proposed that such predictive force control relies on internal representations, i.e., internal models, of the relation between the dynamic of the environment and movement kinematics. Somatosensory and visual feedback plays a primary role in building these internal representations. For instance, it has been shown that manipulation-dependent somatosensory signals contribute to building internal representations of gravity in normal and altered gravitational contexts. Furthermore, delaying the timing of visual feedback of object displacement has been shown to affect GF. Here, we explored whether and the extent to which spatial features of visual feedback movement, such as motion direction, may contribute to GF control. If this were the case, a spatial mismatch between actual (somatosensory) and visual feedback of object motion would elicit changes in GF modulation. We tested this hypothesis by asking participants to generate vertical object movements while visual feedback of object position was congruent (0° rotation) or incongruent (180° or 90°) with the actual object displacement. The role of vision on GF control was quantified by the temporal shift of GF modulation as a function of visual feedback orientation and actual object motion direction. GF control was affected by visual feedback when this was incongruent in the vertical (180°), but not horizontal dimension. Importantly, 180° visual feedback rotation delayed and anticipated GF modulation during upward and downward actual movements, respectively. Our findings suggest that during manipulation, spatial features of visual feedback motion are used to predict upcoming LF changes. Furthermore, the present study provides evidence that an internal model of gravity contributes to GF control by influencing sensory reweighting processes during object manipulation.

## Introduction

A substantial body of evidence indicates that the human ability to perform dexterous manipulation depends on two control modes, known as reactive and predictive (for review see, Flanagan and Johansson, 2002). Both types of control aimed at attaining and maintaining grasp stability (preventing the object from slipping) by modulating digit forces orthogonal to object surface grip force (GF) in relation to inertial force changes load force (LF; Flanagan et al., 1993). Reactive force control is implemented by modulating GF in response to unexpected LF changes detected by tactile afferents (Cutkosky in Uygur et al., 2012; Prescott et al., 2018). This strategy is particularly useful when manipulating an object in novel dynamic contexts, e.g., interacting with unfamiliar objects or responding to external perturbations.

However, it has been shown that through repeated interactions, the central nervous system (CNS) builds internal representations of the new manipulation contexts. These representations are based on sensorimotor mapping combining movement kinematics with context-dependent multisensory signals (Crevecoeur et al., 2010). Internal representations, or models, have been proposed as mechanisms for predictive force control, as they are assumed to capture invariant features of the arm (e.g., dynamic), the object (e.g., size, weight), and the environment (e.g., gravity; Flanagan and Johansson, 2002; Augurelle et al., 2003; White et al., 2005; White, 2015).

Vision and somatosensory feedback associated to movement kinematics are known to play an important role in building and retrieving internal representations for object manipulations. For instance, it has been proposed that GF adjustments associated with new gravitational fields depend on internal representations of the new dynamic of the environment acquired through somatosensory inputs (Augurelle et al., 2003; White et al., 2005; White, 2015). Similarly, online visual feedback has been shown to influence internal representations and predictions of LF fluctuations. Sarlegna et al. (2010) first showed that delayed visual feedback of object motion relative to veridical somatosensory feedback influences grip-LF temporal coupling during a manual tracking task. More recently, van Polanen et al. (2019) reported that the introduction of a visual delay of object kinematics causes a reduction in fingertip force rate while lifting an object. Together, these findings show that visual, as well as somatosensory, feedback of object motion contributes to the acquisition of a new internal representation of the dynamic of the environment, hence influencing predictions of LF changes. These findings emphasize the role of online visual feedback on digit force control and reveal that, during objects manipulation, the temporal characteristics of visual cues of object motion are taken into account for predicting LF modulation. Importantly, this evidence also raises the question of whether spatial features of visual feedback motion, such as movement direction, contribute to predictive digit force control.

The influence of visual feedback directly on motor planning and execution has been previously investigated in the context of goal-directed arm movements (Kilner et al., 2003; Stanley et al., 2007; Sciutti et al., 2012; Toma et al., 2015). Sciutti et al. (2012) studied the effects of a mismatch between the direction of visual feedback motion and actual arm displacement and found that altered visual information along the vertical, but not horizontal axis influenced the temporal patterns of arm kinematics (i.e., asymmetric vs. symmetric velocity profiles, respectively). These findings were interpreted in support of the hypothesized role of visual inputs in making predictions of the effect of gravity acting on the body and the objects, i.e., “visual gravity.” The concept of visual gravity has been supported by several studies suggesting that the sensorimotor system takes into account the effects of gravity experienced through vision, e.g., falling object, to interpret and predict body/object motion features (Runeson and Frykholm, 1981; Maffei et al., 2015). While the role of visual gravity has been already investigated in the context of arm kinematics during pointing and catching (McIntyre et al., 2001; Le Seac’h and McIntyre, 2007; Zago et al., 2008; Sciutti et al., 2012; Toma et al., 2015), its role for digit force control has been overlooked.

**Figure 1:**
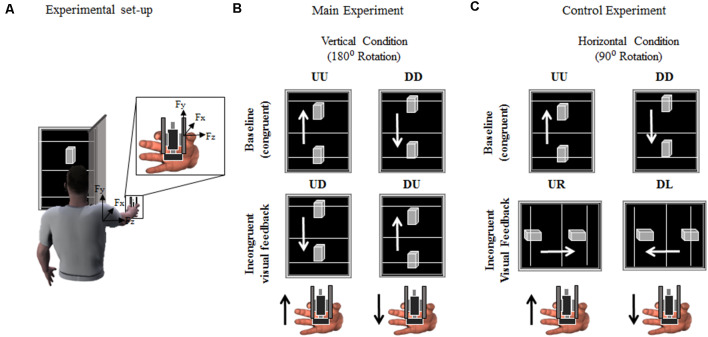
Experimental set-up and protocol. **(A)** Participants were asked to grasp a grip device using a precision grip while performing vertical movements through rotation around the shoulder joint, i.e., shoulder flexion. The gripping device (object) was used to record the index finger and thumb normal and tangential (vertical) forces (F_z_ and F_y_, respectively). Active markers were placed on the shoulder and elbow and wrist joints to track angular excursions. The time-varying position (P_y_) of a marker placed on the object was used to provide online visual feedback of its position on a monitor. **(B,C)** Both Main and Control experiment consisted of two sessions of 54 vertical movements each, i.e., interleaved sequence of 27 upward and 27 downward movements. Visual feedback of object upward or downward vertical displacement was either congruent (Up-Up or Down-Down: UU and DD, respectively) or rotated, i.e., incongruent, 180° (Up-Down or Down-Up: UD and DU; Main Experiment) or 90° (Up-Right or Down-Left: UR and DL, respectively; Control Experiment). Black and white arrows in **(B,C)** indicate object/hand and visual feedback directions, respectively, for each condition and experiment. For clarity, the hand in **(A–C)** is shown from a different perspective relative to the image of the subject holding the object in **(A)**.

The present study aimed at exploring the role of visual gravity and visual feedback of object motion direction for manipulation. We addressed this issue by introducing a spatial mismatch between actual (somatosensory) and visual feedback of object motion direction (180° and 90° rotated) while subjects generated vertical hand-held object movements (Figure 1). This paradigm allowed us to investigate the effects of visual gravity on GF control by providing incongruent visual and somatosensory inputs of the spatial features of object motion, e.g., upward object motion is visually displayed as downward object motion, and vice versa. We reasoned that, if visual inputs of object motion direction contributed to GF control, incongruent visual feedback would influence the timing of digit force modulation relative to congruent feedback conditions. Importantly, if visual gravity was responsible for changes in GF control during incongruent conditions, we would observe upward and downward visual feedback to differentially affect the timing of GF modulation. Similarly, if predictions of LF fluctuations depended on an internal representation of gravity, GF timing associated with actual vertical object movement would be modified relative to baseline when incongruent visual feedback is vertically, but not horizontally oriented.

Our hypothesis is based on previous evidence showing that when visual and somatosensory cues are congruent—i.e., visual feedback provides veridical information about object motion—GF modulation during upward, downward, and horizontal movements exhibit a different temporal pattern. It has been proposed that these direction-dependent differences in digit force modulation depend on predictions of the effect of gravity and inertia associated with the kinetic of the manipulation (Jaric et al., 2005; Zatsiorsky et al., 2005). Importantly, such predictive behavior drives predictions of single LF peaks, and consequent GF modulation, occurring towards the beginning of the end of motion during upward or downward object movements, respectively (Supplementary Figure S1). In contrast, during horizontal movements, an increase in force at the beginning of object motion is maintained until the end of displacement (Flanagan and Tresilian, 1994). Since direction-dependent LF modulation results from the relation among object mass, motion kinematic and gravitational forces, GF control aims at anticipating LF modulation based on direction, object and velocity-dependent predictions of manipulation dynamic, i.e., GF peak anticipates LF peak in both upwards and downwards vertical motion directions (Supplementary Figure S1). Based on this evidence on direction-specific temporal patterns of digit force modulation, we predicted that when visual and somatosensory information about vertical object motion is incongruent—i.e., visual feedback of object motion is 180° rotated with respect to actual object motion—the influence of vision on LF predictions will result in delayed and anticipated GF modulation, relative to congruent condition, for upward and downward actual motions, respectively.

We note that similar to a temporal decoupling (Sarlegna et al., 2010), the introduction of spatial mismatch between actual and visual feedback of object motion direction does not entail a change in GF modulation to maintain grasp stability since the actual task dynamic remains unchanged. Consequently, and based on previous work investigating the effect of visual feedback on GF control and visual gravity in movement planning, we expected to observe direction-dependent changes in the timing of GF modulation rather than GF magnitude.

## Materials and Methods

### Participants

Thirty-three right-handed (self-reported) subjects participated in this study. Nineteen subjects (15 males, mean age = 24.2 years ± 5.1) participated in the main experiment. A second group of nineteen subjects (six males, mean age 26.5 ± 6.2 years) participated in a control experiment. This second group consisted of 14 new subjects and five subjects that also participated in the main experiment. None of the subjects had neuromuscular disorders and all had a normal or corrected-to-normal vision. All subjects were naïve to the purpose of the study and all gave written informed consent according to the declaration of Helsinki. The protocols were approved by the Office of Research Integrity and Assurance at Arizona State University.

### Manipulandum

Subjects were required to grasp a customized grip handle. The design of the gripping device has been described elsewhere (Fu et al., 2010). Briefly, the gripping device consists of two parallel vertical components and equipped with two hidden 6-axis force-torque sensors (Nano-25; ATI Industrial automation, Garner, NC, USA). The vertical bars and the transducers were mounted collinearly to each other on opposite sides of the gripping device (65 mm apart; Figure 1A, inset). The contact surfaces of the bars were covered with 100-grit sandpaper (static friction coefficient range: 1.4–1.5). The total mass of the manipulandum (object hereafter) was 400 g. Digit force and torque data were acquired with a 12-bit A/D converter (PCI-6225; National Instruments, Austin, TX, USA), digitized at 1 kHz, and collected through a custom-designed routine written in LabView (National Instrument, Austin, TX, USA).

### Motion Tracking

Arm and object kinematics were recorded by a motion capture system (eight cameras; frame rate: 120 Hz; spatial resolution: 0.1 mm; Phase Space Inc., San Leandro, CA, USA). Active markers were placed on the top of the object, shoulder (acromion), elbow (epicondylus lateralis), and wrist joint (the styloid process of the ulna). Arm and object kinematic data were collected and stored using a custom design program written in Labview.

### Visual Feedback

The time-varying vertical position of the tracked object’s marker was used to update the position of a 3D object displayed on a monitor (24”; refresh rate: 60 Hz), placed 70 cm from the subject’s eyes (Figure 1A). In the main experiment, the monitor was vertically oriented during both the baseline and the incongruent condition (Figure 1B; see “Experimental Procedures” section). During the Control experiment, the monitor was oriented vertically during the baseline trials and rotated 90° during the incongruent trials (Figure 1C; see “Experimental Procedures” section). Along with the 3D visual feedback of object motion (visual feedback hereafter), three reference lines were projected orthogonally to the long side of the monitor (Figure 1). These lines (15-cm apart) provided a cue for the range of motion subjects were instructed to produce at each trial. In both Main and Control experiments, a screen (Figure 1A) blocked the vision of the object and subject’s hand. The scaling factor between the object displacement and shoulder joint flexion was ≈0.45 (i.e., 30 ± 10 cm object displacement corresponding to a shoulder rotation of 65°± 6°, mean ± SD; across- subjects’ arm length 64.4 ± 3.1 cm). The visual display, force recording from the manipulandum, and motion capture were synchronized using Labview code. The time delay between motion capture of object kinematics and visual rendering was ≈40 ms.

### Experimental Procedures

Subjects were instructed to execute discrete upward and downward arm movements while grasping the object and being comfortably seated on a chair with their right arm extended (Figure 1A). They were required to produce ballistic vertical object motions by flexing and extending the shoulder joint while keeping the elbow fully extended and without moving the wrist. At the beginning of each trial, subjects were required to lift the object as described above such as to position the visual feedback on one of the starting reference lines, i.e., lower or upper line for upward and downward motions, respectively (Figures 1B,C). The arm at starting position subtended a shoulder elevation angle of about −21° ± 1.2° and 1° ± 1.7° (median ± SE) for upward and downward trials, respectively (0° being the arm parallel to the transverse plane). Average shoulder abduction angle was 21° ± 1.2° and 18° ± 2° for upward and downward motions, respectively, where positive values indicate outward deviation from the sagittal plane. The experimenter, after verifying that the subject had held the object at the desired start location, verbally cued the subject to produce a movement aimed at moving the visual feedback display towards the target line, i.e., upper and lower line for upward and downward motions, respectively (Figures 1B,C), and terminate the movement there. While subjects were required to be consistent with the amplitude and velocity of their movements across trials, they were allowed to produce small under- or over-shoots, corresponding to the visual feedback display going below or above the target line, respectively. However, we emphasized prioritizing the ballistic component of the movements rather than the accuracy of stopping the motions at the target line. This was an important component of our design aimed to maximize the occurrence of single-peak object velocity profiles. Trials characterized by multiple velocity peaks were excluded from further analysis. On average, 4 ± 1% (mean ± SD) of the trials collected across subjects and conditions were excluded. Across-subject median ± CI of the object velocity was 1.1 ± 0.03 m/s.

### Main Experiment

During *baseline condition*, subjects were asked to perform arm movements aimed to move the visual feedback from the lower to upper lines (i.e., upward movement, UU) or vice versa (i.e., downward movement, DD; top row, Figures 1B,C). Visual feedback and actual movement direction of the object were congruent, i.e., in the same movement direction. During the *incongruent condition*, subjects were instructed to comply with the same movement instructions described above, being the visual feedback of object movement direction rotated 180° relative to baseline motion. Therefore, the upwards movement of the object resulted in visual feedback of downward movement, and vice versa (UD and DU, respectively; Figure 1B).

### Control Experiment

Subjects assigned to this experimental group performed a baseline condition identical to the main experiment baseline (Figure 1C, baseline). However, during the incongruent condition of the control experiment, subjects were asked to perform vertical object movements, while visual feedback was displayed on a monitor that rotated horizontally relative to arm and object motions (Figure 1C, incongruent visual feedback). Therefore, subjects were provided with visual feedback of object kinematic that was rotated 90° relative to the actual, vertical, object movement direction, i.e., upward and downward object motion was associated with the visual feedback displayed rightward (UR) and leftward (DL), respectively.

In both Control and Main experiments, subjects performed 54 movements for each condition, 27 for each arm movement direction. In each condition, upward and downward movement trials were presented in an alternating fashion. To prevent muscle fatigue, subjects were given a break (30 s) every six trials and between baseline and incongruent conditions (5 min). The first four trials of each condition, two upward and two downward, were used to allow subjects to familiarize themselves with the task and were not analyzed.

### Digit Forces

The 6-axis force/torque sensor on each side of the object measured grip and tangential (vertical) force exerted by thumb and index fingertip (F_z_ and F_y_, respectively; Figure 1A; inset). Force data were filtered with a fourth-order zero-lag Butterworth filter (20 Hz cutoff frequency). Data analysis was performed on the mean normal force exerted by the thumb and index finger (GF), and on the sum of the recorded tangential forces (*LF*). We averaged grip and LFs exerted during object hold against gravity across 250 ms before movement onset (*static* grasp component) and subtracted these data from the grip and LFs exerted during the movement (*dynamic* grasp component). As done in previous work, this decomposition in static and dynamic components allows measurement of grip and LF modulation while taking into account within- and between-subjects variability (Crevecoeur et al., 2009, 2010). The dynamic grasp component was normalized in time and magnitude.

### Movement Kinematic and Dynamic

Arm joints and hand-held object positions were low-pass filtered with a second-order, 5 Hz cut off, zero-lag Butterworth filter. Position data were then used to estimate object linear velocity and acceleration. To compensate for inter-individual differences in movement amplitude dependent on different subjects’ arm length, we measured the amount of movement variation across conditions by using the coefficient of variation (CV). Similar, to a previous study (Crevecoeur et al., 2010), this parameter allows quantification of participants’ accuracy while performing hand-held object motion across visual feedback conditions.

Arm joint position, velocity, and acceleration were used to estimate shoulder and elbow angle joint rotations and total torque profiles (Supplementary Figure S2, *Joint Net torque*) employing a kinematic and kinetic model incorporating geometrical and inertial parameters of subjects’ arm segment (see “Kinematic and Kinetic Model of the Arm” section). Total (net) shoulder and elbow joint torques were estimated *via* recursive Newton Euler calculation considering inertial, Coriolis, centripetal and gravitational components (D’Andola et al., 2013; Russo et al., 2014; Toma and Lacquaniti, 2016). This analysis aims at detecting any effect of visual feedback rotation on shoulder-elbow joint coupling and arm dynamics. Importantly, while the analysis performed on object velocity provides information about the relation between arm kinematics and digit forces, joint torque timing could also provide insights on the arm-hand-GF relation across conditions.

For each trial, movement onset and offset were defined as the time when the object linear velocity was greater (motion onset) and lower (motion offset) than 2% of the peak velocity, respectively (Supplementary Figure S2, vertical dashed lines). To match movement profiles with force data, kinematic data were linearly interpolated from 120 to 1,000 Hz. Position, velocity, acceleration, angles, and torque profiles were then time normalized. The normalization guarantees that velocity and acceleration profiles are independent of the distance traveled the movement speed, and the movement duration (Gaveau and Papaxanthis, 2011). Consequently, and similarly to force profiles, time-to-peak velocity and time-to-peak torques were expressed in terms of normalized, % of, movement duration.

#### Kinematic and Kinetic Model of the Arm

The kinematic model (adapted from Russo et al., 2014) consists of a chain of seven articulated links. Each link is defined by four parameters: length (*a*), twist (α), offset (*d*) and joint angle (*θ*; Supplementary Table S1) describing the position and orientation of a Cartesian reference frame fixed on each link. Following the D-H (Denavit-Hartenberg) convention (Hartenberg and Denavit, 1955), the rotation axis of each joint is the z-axis of the preceding link in the chain. The x-axis in each frame is directed as the normal between the rotation axis of that frame and the rotation axis of the next frame (see Russo et al., 2014 for details on the homogenous transformation matrix). The model describes four rotational degrees of freedom (DoF) of the arm, three rotation DoFs at the shoulder, i.e., adduction, flexion and external rotation, and one rotation DoF at the elbow, i.e., elbow flexion (Supplementary Figure S3). Three translational DoFs of the shoulder were also considered. The shoulder joint was modeled as a spherical joint, i.e., rotation axes of the three joints intersect at a single point (Russo et al., 2014). Joint angles *q* extracted from the model, joint velocities $\sqrt{\dot{q}}$, and accelerations $\sqrt{\ddot{q}}$ obtained from markers dataset were used to estimate the torque profiles actively generated by the subjects *via* recursive Newton-Euler calculation (*rne* function of Matlab Robotics toolbox; Corke, 1996). Total torque, τ, was computed using the Matlab Robotics toolbox as follows:

(1) $$\sqrt{\tau =M(q)\ddot{q}+C(q,\dot{q})\dot{q}+G(q)}$$

where *M* is the matrix of principal inertia moments, *C* is the Coriolis and centripetal torque, and *G* is the gravitational torque. For each time sample and joint angle, a vector between two markers aligned with the axis of the limb segment defining the rotation of that joint was computed first (i.e., shoulder and elbow markers for shoulder abduction and shoulder flexion, elbow and wrist markers for shoulder external rotation and elbow flexion). Then the associated angle was computed for each defined limb segment as $\sqrt{\tan^{-1}(y/x)}$, where *x* and *y* are the coordinates of the vector in the reference frame associated with the joint rotation axis z (Russo et al., 2014). Joint angles definitions are depicted in Supplementary Figure S3. Examples of the joint elbow and shoulder flexion torques from a representative subject are shown in Supplementary Figure S2. Differently from Russo et al. (2014); we estimated mass, the center of mass, and inertia tensor of each link using direct measurements of individual limbs’ length as well as girth of the upper arm, forearm, and hand (Zatsiorsky et al.’s equations and De Leva’s adjustments; de Leva, 1996; Zatsiorsky et al., 1990). The kinetic model was then developed by adding the inertial parameter to each link. As in Russo et al. (2014), the estimated position of the center of mass of the hand and instrumented object coincided, thus the mass of the manipulandum (400 g) was added to the mass of the hand. The D-H parameters of the generic arm model are shown in Supplementary Table S1. The range of geometric and inertial parameters extracted across subjects is shown in Supplementary Table S2.

#### Parameters Difference and Two-Factorial Design

To determine the influence of visual gravity on GF control, we tested the hypothesis of whether online visual information of hand-held object displacement drives direction-dependent changes in GF control. We addressed this question by focusing on seven variables: (1) coefficient of variation (CV) of arm movement amplitude; (2) time-to-peak object velocity; (3) grip and LF maxima; (4) time-to-peak grip and LF; (5) time-to-peak grip and LF rate; (6) time-to-peak shoulder and elbow torque; and (7) grip-LF coupling. These variables were extracted from the time-normalized movement and force profiles from baseline (congruent) and incongruent trials. At the individual-subject level, we quantified the effect of visual feedback rotation by subtracting incongruent trials data from baseline trial data, i.e., Δ (variable). At the population level, we considered the across-subjects median of each Δ variable calculated in each trial, and for upward and downward movements separately. Consequently, the effect of the visual feedback rotation across subjects was defined as the overall distribution of Δ-variables for each movement direction. By using the difference between baseline and incongruent data, we could test the main effect of visual feedback rotation, i.e., Δ-variables that were significantly different than zero (effect of *Rotation*) in object movement kinematic, arm dynamic and digit forces. The effect of *Direction* was assessed by testing the direction of changes elicited by the visual feedback rotation, i.e., positive or negative Δ-variables. We also assessed the interaction between the direction of visual feedback rotation and the direction of the actual object motion, i.e., the statistical significance of the difference between the effect of visual feedback rotation observed during upward and downward motions (effect of *Rotation* × *Direction*). We chose to use non-parametric statistics because some participants’ data-set exhibited non-normal distribution. Also, due to the presence of the outlier, the median of the data distribution was considered a better descriptor of our data than the mean.

Given our use of a two-factor design with repeated measures, we could not perform non-parametric statistics assuming independance of observations (e.g., Kruskal-Wallis test) and/or testing for only one factor (e.g., Friedman Anova). Therefore, we assessed the main effect of rotation on each of the above-mentioned variables by performing a one-sample non-parametric Wilcoxon sign rank analysis aimed at testing for zero medians of the Δ variables distribution. The main effect of direction was assessed by one-tailed non-parametric analysis testing for median either higher or lower than zero. We assessed the interaction effect between *Rotation* and *Direction* using a paired-sample Wilcoxon sign rank tests comparing UD with DU Δ-variables. The observation of statistically significant interactions indicates a direction-dependent effect of visual feedback rotation on the variable being tested, i.e., visual feedback influences GF modulation differently during upward vs. downward object motion. We corrected the statistics using Bonferroni corrections by dividing the critical *p*-value by the number of comparisons, i.e., *p* < 0.02. We also computed the effect size *r* as the ratio between the non-parametric test output and the squared-root of the number of samples: *r* = Z-statistics/$\sqrt{N}$ (Rosenthal, 1994).

#### Grip-Load Coupling

Grip-LF coupling was assessed using cross-correlation analysis between grip and LF profiles (Flanagan and Wing, 1997; Sarlegna et al., 2010). This analysis aimed at quantifying whether and to what extent GF profiles shifted in time, relative to LF, as a function of visual feedback and actual object motion direction. Positive and negative lags (ms) denote GF leading and lagging LF, respectively. Importantly, this analysis considers the entire time course of GF and LF as opposed to discrete time-points (i.e., time-to-peak force and peak force rate), hence providing a more reliable measure of the temporal shift of the force profiles.

We further quantified the effect of visual feedback on digit force modulation by quantifying the relation between GF and LF in each experimental condition. Specifically, we computed the correlation coefficients, and related statistics, describing the coupling between the time-to-peak grip and LF. Similarly, we performed classical least-square linear regression (Crevecoeur et al., 2010) on the same dataset to obtain slope regression values, and their associated statistics, for each condition. We first obtained the statistics on the correlation coefficient and regression slope for each condition separately. Then, we compared the change in slope values between baseline and incongruent trials by obtaining their distributions, i.e., 200 iterations bootstrap sampling, and performed a Wilcoxon Signed rank test.

#### Quantification of the Effect of Visual Feedback Direction on Digit Force Control

We quantified the amount of digit force modulation associated with the visual input rotation (visual effect, VE) during UD trials using the following equation (Toma et al., 2015):

(2) $$\sqrt{VE_{\text{cnfUD}}}=\sqrt{\frac{BSL_{\text{uu}}-CFL_{\text{ud}}}{BSL_{\text{uu}}-BSL_{\text{dd}}}}\cdot 100$$

Similarly, during DU trials we used:

(3) VEcnfDU=(BSLdd−CFLduBSLuu−BSLdd)⋅100

Where $\sqrt{BSL_{\text{uu}}}$ and $\sqrt{BSL_{\text{dd}}}$ are the time-to-peak grip (or load) force exhibited during baseline trials, and $\sqrt{CFL_{\text{ud}}}$ and $\sqrt{CFL_{\text{du}}}$ are the time-to-peak grip (or load) force exhibited during UD and DU incongruent conditions, respectively. Accordingly, the absolute maximum shift (100%) is observed whether upward (UD) and downward (DU) incongruent trials exhibited a time-to-peak GF equal to downward and upward baseline values, respectively. Thus, VE quantifies the extent to which visual input of hand-held object direction influences both load and GF modulation during upward and downward object/hand movements.

## Results

### Effect of Visuomotor Conflict on Arm Kinematics

**Figure 2:**
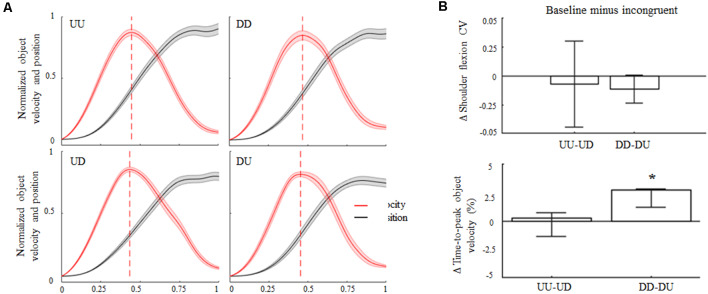
Object kinematics. **(A)** Across subjects median ± SE of velocity (red trace) and position (black trace) profiles of object displacement during congruent (UU, DD) and incongruent (UD, DU) conditions. For each subject’s dataset, velocity and position profiles were normalized in time and amplitude for their maximum values exhibited in the same condition. Profile onset and end were defined as the time when the object linear velocity was greater and lower than 2% of peak velocity, respectively. In each plot, vertical dashed lines denote across-subjects median of time-to-peak velocity. **(B)** Median ± 95% confidence intervals (CI) computed from the difference between arm movement coefficient of variations (ΔCV) exhibited during congruent and incongruent trials (upper plot). Positive and negative Δ shoulder flexion CV values indicate lower and higher variability in movements amplitude during incongruent trials relative to congruent conditions, respectively. Positive and negative Δ time-to-peak object velocity medians and CI (bottom plot) indicate that peak velocity was anticipated or delayed for baseline during incongruent trials, respectively. *Denotes median value statistically significant than 0. Note that the vertical dashed lines in **(A)** denote time-to-peak velocity of the across-subjects median of the entire velocity profile, whereas median Δ values across all subjects and trials in **(A)** were obtained by only considering time-to-peak velocity.

Subjects complied with the instruction of keeping the arm straight, as denoted by negligible elbow flexion angles both during baseline and incongruent trials for upward and downward object motions (overall CI: 0.7°–5.3°). During baseline and incongruent trials subjects produced similar median shoulder elevations for upward and downward object motions (≈65°± 6°). Across trials and conditions, subjects exhibited more variability in performing upward movements’ amplitude rather than downward motions (Figure 2B, top). Nevertheless, we found no statistical differences in the coefficient of variation (CV) in movement amplitude from baseline and incongruent conditions for upward or downward movements. In agreement with previous studies (Papaxanthis et al., 1998; Gaveau and Papaxanthis, 2011), time-to-peak hand/object velocity occurred earlier in UU than DD trials (47% and 51% of total movement time, respectively; Figure 2A). No main effect of 180° visual feedback rotation was found during upward object motion as the time-to-peak velocity during UD trials did not exhibit statistically significant changes relative to baseline (median 47%; CI: 46%, 47%; *z* = 2.45, *p* = −0.23). In contrast, upward-rotated visual feedback during downward motion (i.e., DU) caused a significant reduction in the time-to-peak velocity with respect to baseline (48%; CI: 47%, 48%; main effect, *z* = 3.24, *p* < 0.01, effect size *r* = 0.65; Figure 2B, bottom).

### Visual Feedback Rotation: Modulation of Digit Force Amplitude and Timing

Overall, the rotation of the visual feedback did not elicit the main effect of modulation of LF maxima with respect to baseline. Specifically, Δ variable associated with LF maxima exhibited during upward and downward movements was not significantly different than 0 (*z* = 0.39, *p* = 0.69 and *z* = −0.44, *p* = 0.66, respectively). Conversely, GF maxima were significantly modulated during downward incongruent trials, but not during incongruent upward object motions (*z* = −1.46, *p* = 0.14). Specifically, during DU trials visual feedback direction elicited GF increase of about 16% relative to baseline (main effect, *z* = −3.1, *p* < 0.01).

**Figure 3:**
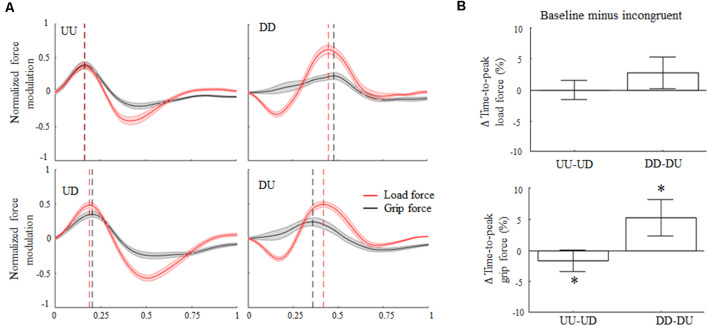
Digit force modulation. **(A)** Across-subjects median ± SE of load (red trace) and grip (black trace) time course of force during object displacement. For each subject, the static force component was subtracted from each profile (see text) and normalized in time and amplitude concerning their maximum values exhibited in the same condition. Profile onset and end were defined following movement onset and end, i.e., 2% of velocity profile peak. Black and red vertical lines represent across subjects median time-to-peak grip and load force (LF), respectively. **(B)** Median and CI values of time-to-peak load and grip force (GF; top and bottom plot, respectively) computed as the difference between baseline (congruent) and incongruent trials. An asterisk denotes median values significantly different than zero (Wilcoxon sign-rank test, *p* < 0.05). Note that, as in Figure 2; the vertical dashed lines in **(A)** denote time-to-peak force of the across-subjects median of the entire force profiles, whereas median Δ values across all subjects and trials in **(B)** were obtained by only considering the time-to-peak forces.

Concerning force peak timing, during baseline conditions the time-to-peak LF occurred early in the movement for upward motions (median 31%; CI: 31%, 32%) and later for downward movements (74%; CI: 72%, 75%). The 180° rotation of the visual feedback elicited relatively small changes in the time-to-peak LF (Figure 3B, top) for both movement directions. Specifically, the median of the Δ time-to-peak LF was 0% (CI: −1%, 1%) for upward and 2.7% (CI: 0.9%; 4.5%) for downward movements, respectively. Indeed, no main effect of visual feedback rotation was observed on the time-to-peak LF modulation for upward or downward motions (*z* = −1.28, *p* = 0.2 and *z* = 2.11, *p* = 0.03, respectively). However, during incongruent downward object motions, subjects exhibited a weak tendency to anticipate peak LF occurrence relative to baseline, i.e., positive Δ values (effect size *r* = 0.44; Figure 3A, right column, and Figure 3B, top). Note that the description of the effect of visual feedback on the time-to-peak LF shown in Figure 3A slightly differs from the effect shown in Figure 3B, i.e., only the former condition shows an increase in time-to-peak LF during UD. This difference depends on the fact that, while in Figure 3A the time-to-peak force was calculated from the entire force profiles (median across subjects) the effect described in Figure 3B shows values obtained by considering only one time-point, i.e., time-to-peak force (see “Grip-Load Force Coupling” section for results of the analysis of whole force profiles and Supplementary Figures S4, S5 for individual results).

The time-to-peak GF exhibited during both UU and DD trials reproduced an asymmetric temporal pattern similar to that one observed for the LF profiles, hence suggesting grip-LF coupling. Specifically, time-to-peak GF median was 37% (CI: 36%, 38%) for UU and 69% (CI: 67%, 70%) for DD, respectively. Importantly, the main effect of 180° visual feedback rotation on GF timing was found for both movement directions. Specifically, during UD incongruent trials the time-to-peak GF occurred later in the movement with respect to UU trials, i.e., 38%; CI: 37%, 39%, whereas it occurred earlier in the movement for DU trials for downward baseline (65%; CI: 63%, 67%). Δ values describing the effect of visual feedback rotation on the time-to-peak GF were statistically different than 0 for both upward (*z* = −2.54, *p* = 0.01, effect size *r* = 0.51) and downward (*z* = 3.37; *p* < 0.01, effect size *r* = 0.67) movements (Figure 3B, bottom). A statistically significant *Rotation × Direction* interaction was also found (*z* = −3.37, *p* < 0.01, *r* = 0.67), suggesting a direction-dependent effect of visual input direction on GF timing modulation.

The same appraisal of digit peak force timing across conditions was repeated considering the group of subjects who participated exclusively in the main experiment (*n* = 14). This further analysis aimed at verifying whether the presence of five subjects performing both main and control experiments (i.e., repeated-measure participants) might have contaminated our findings. The exclusion of the repeated-measure participants from the analysis did show any significant difference relative to the consideration of the entire subjects’ sample, hence excluding potential contamination of our mixed-design. Specifically, the overall digit force modulation and time to peak force statistics associated with the reduced sample size resembled the entire sample behavior, i.e., *n* = 19 (Supplementary Figure S6). The delta parameters describing the effect of visual feedback rotation on the time-to-peak LF was found again not statistically different than zero during upward (*z* = −2.16; *p* = 0.03) and downward motions (*z* = 2.00; *p* = 0.04). In contrast, the statistically significant modulation of GF timing persisted for both UD (*z* = −2.38; *p* = 0.017; *r* = 0.47) and DU (*z* = 3.67; *p* < 0.01; *r* = 0.74) incongruent conditions after removing the repeated-measures participants.

The analysis performed on the entire sample group revealed that neither grip nor load peak force rate timing exhibited a main effect of 180° visual feedback rotation (Supplementary Figure S7). Across subjects’ median ± CI of Δ time-to-peak LF rate was 0.0 (CI: −0.006, 0.0071; *z* = −0.31; *p* = 0.75) for UD and 0.01 (CI: 0.002, 0.03; *z* = −1.17; *p* = 0.24) for DU trials. Similar to LF, no main effects of visual feedback rotation was observed for Δ time-to-peak GF rate, i.e., 0.0 (CI: −0.014, 0.012; *z* = −0.09; *p* = 0.92) for UD and 0.04 (CI: −0.01, 0.07; *z* = 1.79; *p* = 0.07) for DU trials. Across conditions, the inter-quantile range (IQR) of the distributions of the Δ time-to-peak GF was characterized by consistently higher variance than the GF rate (Supplementary Figure S8) for UD (0.03 and 0.05, respectively) and DU (0.09 and 0.13, respectively) motions. Moreover, despite the lack of the main effect of visual feedback rotation on Δ peak force rate timing, during incongruent conditions both time-to-peak GF and GF rate tended to negative and positive Δ values for UD and DU conditions, respectively (Supplementary Figure S8). These findings indicate that the effect of visual feedback rotation on the time-to-peak GF rate was contaminated by higher variability than the time-to-peak GF.

### Grip-Load Force Coupling

**Figure 4:**
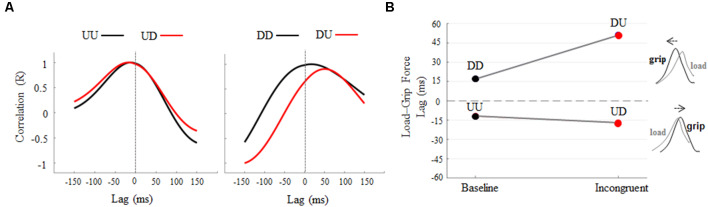
Cross-correlation analysis: load-GF-time lag across conditions. **(A)** Each curve describes lag and correlation between grip and LF profiles obtained during baseline (black) and incongruent (red) conditions. Cross-correlations curves were obtained from across subjects’ median force profiles (Figure 3A). Positive and negative lag values indicate that GF precedes or follows LF, respectively. **(B)** The opposite effect of 180° visual feedback rotation on Load-Grip lag, i.e., delayed and anticipated GF modulation for LF during upward and downward object movements, respectively.

Cross-correlation analysis revealed an increase, relative to baseline, of negative lag between grip and LF profiles during the UD condition (Figure 4A, left). An increase of positive lag was found when subjects produced downward object movements while visual feedback of motion direction was rotated 180°, i.e., DU (Figure 4A, right). Overall, 180° rotation elicits opposite temporal shift of GF modulation relative to LF (Figure 4B) during upward and downward object motions (Supplementary Figures S9, S10 for individual results). In particular, rotation of visual feedback motion during upward movements elicited a delay in GF modulation, relative to LF, of 5 ms, being −12 ms and −17 ms the grip-load lag exhibited during UU and UD, respectively. A higher effect of visual feedback rotation was observed during downward motions, where GF modulation was anticipated 34 ms relative to LF, the grip-load lag being 17 ms and 51 ms during DD and DU, respectively. Together, these findings in addition to the observation of no main effect of visual feedback rotation on LF timing, i.e., Δ time-to-peak LF and time-to-peak LF rate not statistically different than 0, indicate that the grip-load lag was due to a temporal shift of the GF profiles rather than LF. The same analysis performed on the 14 subjects who only participated in the main experiment did not reveal any relevant difference in the grip-LF coupling behavior observed in the whole participant’s sample (Supplementary Figure S11). In particular, the same amount of delay was observed in GF modulation, i.e., −10 and −15 ms, during UU and UD conditions respectively. On the other hand, we observed a slight reduction of grip-load lag during downward motion, i.e., being 13 ms and 39 ms the grip-load lag exhibited during DD and DU, respectively. Similarly to the time to peak force analysis, this appraisal confirms that the introduction of a group of subjects participating in both control and the main experiment did not contaminate our main findings.

**Figure 5:**
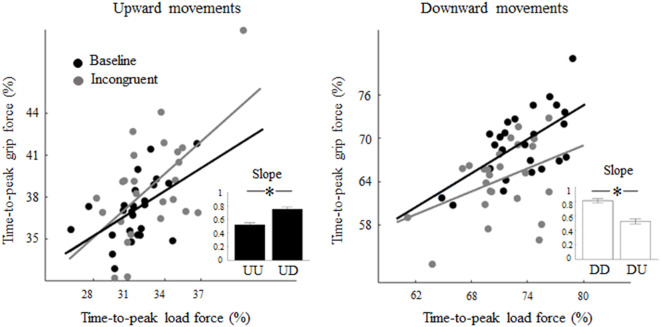
Effect of visual feedback rotation on grip-LF coupling. Each data point is the median time-to-peak grip and LF from each trial computed across all subjects for upward and downward movements (left and right plot, respectively). Black and gray dots are baseline and incongruent (180°) trials, respectively. Note that increase and decrease in slope values relative to baseline indicate that grip-LF coupling during incongruent conditions was shifted earlier and later in time, respectively, relative to congruent conditions. In each plot, the inset depicts median ± CI of slopes distribution obtained through bootstrap sampling (200 iterations; see text for details). *p < 0.05.

Regression analysis performed on the relation between the time-to-peak grip and LF exhibited in each condition showed significant correlation, i.e., Pearson’s *r* = 0.52 (*p* < 0.01) and slope 0.61 (*p* < 0.01), for both upward and downward baseline motions, respectively (black regression lines, Figure 5). While the exposure to a visual feedback rotation did not affect the correlation between grip and LF timing in UD trials (*r* = 0.57, *p* < 0.01), it affected the grip-load coupling in DU trials thereby resulting in non-significant correlation (*r* = 0.39, *p* = 0.05). Importantly, the slope of the linear regression showed a statistically significant increase (slope distributions obtained by bootstrapping) from 0.54 (UU) to 0.76 (UD) during upward movements (*z* = −4.46; *p* < 0.01; *r* = 0.32; Figure 5, inset, black bars). A similar, but opposite change in the coupling of the grip-load peak force was observed in DU trials where the slope exhibited a reduction from 0.79 in DD to 0.53 in DU trials (*z* = 7.27; *p* < 0.01; *r* = 0.51; Figure 5, inset, white bars).

### Visual Effect as a Function of Feedback of Object Motion Direction

**Figure 6:**
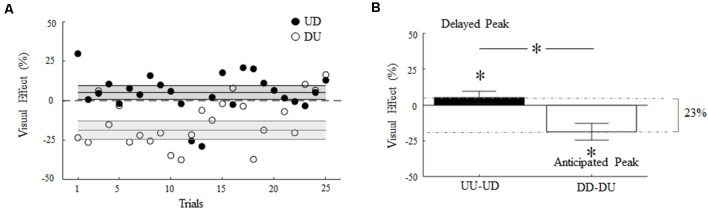
Visual effect of 180° rotation of visual feedback of object motion. **(A)** Percentage change of time-to-peak GF relative to baseline as a function of the trial during UD and DU conditions (filled and empty symbols, respectively). Each symbol denotes the median value across subjects and trials. **(B)** Median and CI of time-to-peak GF shift shown in **(A)**. Asterisk denotes median values significantly different than zero (Wilcoxon sign-rank test, *p* < 0.05). Asterisk between bars indicates a statistically significant interaction between feedback rotation and movement direction. In **(A)** and **(B)**, non-zero values denote a shift of the time-to-peak GF, where positive and negative values denote delay and anticipation relative to baseline, respectively.

Figure 6A depicts the main effect of visual feedback rotation on digit force timing as a function of the trial. In both incongruent conditions, visual feedback rotation caused a transient increase or decrease (filled and empty symbols, respectively) of time-to-peak GF relative to UU and DD baseline (horizontal dashed lines), respectively. This suggests that visual feedback of object motion direction systematically influenced gripping force timing throughout the experiment, although the effect varied across trials.

Overall, visual feedback rotation elicited an absolute shift of the time-to-peak GF from baseline (Visual Effect, VE; see Eqs. 2 and 3) of about 23% (Figure 6B). Rotated visual feedback during UD and DU conditions elicited a median change of 5% (CI: 0.8, 9.5%) and 18% (CI: 12.8, 24.4), respectively, in the time-to-peak GF relative to absolute maximum shift (Figure 6B). Importantly, the direction of these changes appeared to depend on the interaction between the 180° visual feedback rotation and the actual direction of object motion. The time-to-peak GF was delayed in UD trials (VE significantly greater than 0; *z =* 2.43, *p =* 0.01, effect size *r* = 0.49) and occurred earlier in DU trials relative to baseline (VE significantly smaller than 0; *z* = −3.29, *p* < 0.01, effect size *r* = 0.66).

The observation of the influence of visual feedback rotation on peak LF timing, although not statistically significant (see “Visual Feedback Rotation: Modulation of Digit Force Amplitude and Timing” section), may indicate that the main effect observed on the time-to-peak GF during DU trials was a by-product of the induced changes in the time-to-peak LF (Figure 3B, top). To assess this possibility, we subtracted the VE observed in DU trials on LF timing from the VE on GF timing. If the VE observed on GF were exclusively dependent on a shift in LF timing, this subtraction should result in a VE approaching zero, hence resulting in not a statistically significant effect. In contrast, we found that although the subtraction reduced the overall VE (median 9.4%; CI: 4.7%, 14.2%), the effect of the visual feedback on GF timing remained significantly greater than 0 (*z* = 3.26, *p* = 0.001, effect size *r* = 0.62).

Our appraisal of the potential contamination of our mixed-design on VE confirmed our main results (Supplementary Figure S12). Indeed, the removal of the five repeated-measure participants led to a slight increase of the VE observed overall, i.e., 29%, and across motion conditions, i.e., UD: median 7%, CI: 1.2%; 3.2%; DU: median −21%, CI: −14.9%, −28.19%. Importantly, the effect of visual feedback rotation on the modulation of GF timing was still statistically significant for upward (*z* = 2.4, *p* = 0.016, effect size *r* = 0.48) and downward (*z* = −3.7, *p* < 0.01, effect size *r* = 0.74) motions (and after DU subtraction of the VE observed in DU trials on LF timing, i.e., DU: median −14.5%, CI: −8.4%, −19.19%; *z* = −3.5, *p* < 0.01, effect size *r* = 0.7).

### Visual Feedback Rotation and Arm Dynamics

The analysis of the time-to-peak shoulder and elbow torque confirmed the direction-dependent asymmetry observed during baseline trials for arm kinematics and digit force modulation. Specifically, for baseline condition peak shoulder and elbow torque occurred at 41% (CI: 39, 42) and 40% (CI: 39, 42) of upward total motion duration, respectively, and 55% (CI: 53, 57) and 52% (CI: 50; 54) of downward motions. Importantly, we did not observe a main effect of visual feedback rotation on the time-to-peak joint torques (*p* > 0.05). Furthermore, baseline and conflict conditions were not characterized by different time-lag between the shoulder and elbow time-to-peak torque (*p* > 0.05). These findings suggest that the timing of the arm movement dynamic was not influenced by 180° rotation of the visual feedback of object motion.

### Arm Kinematics and Digit Force Control in Response to 90° Visual Feedback Rotation

**Figure 7:**
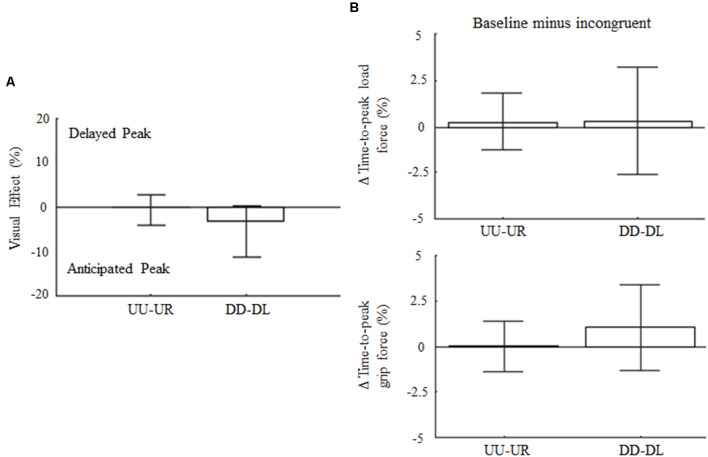
Influence of 90° rotation of visual feedback of object motion on force peak timing. **(A)** Median ± CI of percentage change of time-to-peak GF during incongruent trials relative to baseline. **(B)** Median and CI values of time-to-peak load and GF (top and bottom plot, respectively) computed as for 180° dataset and shown in the same format as in Figure 3B.

The control experiment was designed to cross-validate the hypothesis that direction-dependent modulation of GF timing is not elicited by any incongruent visual feedback, but rather it resembles specific predictions of LF changes associated with the expected effect of gravity based on visual inputs of motion direction, i.e., visual gravity. For the 90° visual feedback rotation, upward and downward arm motions associated to rightward (UR) and leftward (DL) visual feedback, respectively, did not affect movement amplitude variability nor Δ variables with respect to baseline (*z* = 0.36, *p* = 0.72 and *z* = 1.84; *p* = 0.19, respectively). Specifically, no main effects of visual feedback rotation was found on the time-to-peak velocity for upward and downward motions (*z* = −1.33, *p* = 0.55 and *z* = −0.7, *p* = 0.95, respectively). Visual effect and peak LF timing (Figures 7A,B, top) were not affected by the 90° leftward and rightward visual feedback rotation (*z* = −0.58, *p* = 1.68 and *z* = −0.17, *p* = 2.58, for UR and DL, respectively). Importantly, no main effect of 90° visual feedback rotation was associated to GF peak timing, whose median Δ variable values were 0% for UR and DL movements, i.e., *z* = −2.65, *p* = 0.15 and *z* = −1.5, *p* = 0.4, respectively (Figure 7B; bottom). Force profiles and VEs extracted by merging the data from all subjects and trials (median ± SE) are provided as Supplementary Data (Supplementary Figure S13). As for the main experiment analysis, VEs and modulation of time-to-peak forces were re-calculated by considering only those subjects who participated exclusively in the control experiment. This set of analyses performed on 14 subjects confirmed the absence of effect of 90° visual feedback rotation on digit force timing (Supplementary Figure S14).

The analysis performed on the entire sample (*n* = 19) on LF maxima revealed a main effect of visual feedback 90° rotation during DL trials (12% increase; *z* = −2.7, *p* = 0.01). Finally, GF maxima were influenced by 90° visual feedback rotation in both conflict conditions (21% decrease and 22% increase for UR and DL, respectively; *p* < 0.01). The *Rotation × Direction* interaction effect was also found (*p* < 0.01), indicating that the GF magnitude was differentially modulated under UR and DL conditions. The observation of the main effect of 90° visual feedback rotation on grip and LF maxima, but not on their time-to-peak, excludes the possibility that the shift in GF timings observed in the main experiment might have been due to changes in digit force magnitude.

## Discussion

The present work investigated whether visual cues of object motion contribute to predictive GF control during manipulation. We addressed this question by rotating 180° (main experiment) or 90° (control experiment) the visual feedback of object motion and quantified the effects of incongruency between visual and somatosensory inputs by comparing them to a baseline condition (congruent feedback). We tested the hypothesis that spatial features of visual feedback kinematics, such as motion direction, would elicit prediction of the timing of LF fluctuations based on an internal model of gravity, i.e., visual gravity. Accordingly, we expected that the timing of digit force modulation associated to an actual vertical movement: (1) would be influenced by incongruent visual feedback motion direction relative to congruent feedback conditions; (2) would be differently modulated for incongruent upward and downward visual inputs direction; and (3) would be modified relative to baseline when incongruent visual feedback is vertically, but nor horizontally oriented. Our results confirmed our expectations by showing that a mismatch between visual and somatosensory signals of object motion direction caused changes in the timing of GF modulation. Importantly, we observed a temporal shift towards the time that is expected from predictions of LF changes based on the visual object motion direction. In particular, we observed that time-to-peak GF and grip-LF coupling was differentially modulated with respect to whether visual feedback of object motion was upward or downward displayed relative to actual object motion. Furthermore, the results from the control experiment cross-validated our hypothesis of the contribution of visual gravity in GF modulation, since incongruent visual feedback oriented along the horizontal axis did not influence GF timing. We have also shown that the effects of visual motion rotation affected GF, but not shoulder or elbow joint torques. Together, our findings extend the role of online visual feedback for predictive force control mechanisms for manipulation from the temporal to the spatial domain. We discuss our results in the context of multi-sensory integration and visual gravity underlying predictive control mechanisms during object manipulation.

### Visual Gravity Contributes to Anticipatory Grip Force Control

By decoupling visual and somatosensory feedback associated with vertical object movements, we found a visual-induced modulation of the GF timing. Specifically, during the upward incongruent condition (hand-held object moves upward, visual feedback moves downward; UD), subjects shifted peak GF later than baseline whereas, for the opposite incongruent context (DU), subjects shifted peak GF earlier. These findings support our hypothesis that visual feedback of object motion direction drives GF modulation towards the time-to-peak load expected based on the direction of visual feedback of object motion. Our hypothesis of visually-induced predictions about the timing of vertical forces acting on the object is consistent with the concept of visual gravity (McIntyre et al., 2001; Angelaki et al., 2004; Rosenberg and Angelaki, 2014). Previous work revealed that the CNS builds internal representations of the effect of gravity on an ongoing action (e.g., hand or object trajectory, time to contact, motion duration) based on visual motion cues (Pozzo et al., 2006; Zago et al., 2008; Toma et al., 2015). Here we provide novel evidence of the role played by visual gravity in predictive digit force control during manipulation. Specifically, we found that visual feedback of object motion direction significantly influenced time-to-peak GF (Figure 3B, bottom), despite the veridical sensing of LF through somatosensory feedback (Figure 3B, top). Importantly, the direction-dependent effect was found when visual feedback was rotated 180° (Figures 3, 6), but not when it was rotated 90° (Figure 7 and Supplementary Figure S10). We note that, similar to a temporal decoupling of sensory signals (Sarlegna et al., 2010), since the actual task dynamic remains unchanged, the introduction of spatial mismatch between actual and visual feedback of object motion direction does not entail changes in GF modulation aimed at preserving grasp stability.

In contexts of congruent sensory signals, horizontal and vertical movements are characterized by different timing of inertial force changes and associated with direction-dependent grip adjustments aimed at anticipating the different effects of gravity acting on the object. Gravity-related predictive GF control has been described in previous work where upward and downward object motions are characterized by very distinct single GF peak timing, whereas horizontal movements are characterized by a peak elevated throughout the entire motion (Flanagan and Wing, 1993; Flanagan et al., 1993). Thus, consistent with previous evidence, we propose that in scenarios where vision and somatosensory signals are incongruent, GF is modulated according to expected LF changes resulting from integrating visual and somatosensory inputs. Following this interpretation, vertical object motion associated with horizontal visual feedback rotation would influence grip modulation timing to a less extent, and only one direction (e.g., either postponed or anticipated), relative to the vertical incongruent context. Moreover, in agreement with previous work investigating the effect of visual feedback on GF control and visual gravity in movement planning, we found visual inputs to elicit direction-dependent changes in the timing of GF modulation, but not in GF magnitude.

Similar to previous work investigating the direction-dependent effects of visual gravity during pointing movements (Sciutti et al., 2012), our study was designed to test more than one visuomotor contexts, i.e., interleaved upward and downward trials. Therefore, our protocol might have contributed to the across-trial variability of the effect of vision on GF timing. Specifically, we interpret this variability as mostly due to opposite temporal shifts of time-to-peak GF elicited by interleaved upward and downward visual conflict trials. At the same time, one could have expected subjects to habituate to repeated exposure to rotated visual feedback. Surprisingly, this did not happen, as the overall effects of visual conflicts did not decay throughout the experiment (Figure 6A). Such lack of decay, as well as the differential modulation of the time-to-peak force timing during upward, downward (Figure 6B), and 90° (Figure 7) visual feedback rotation, rule out the possibility that our results could have been due to a “default” GF correction strategy employed for any novel environment or incongruent context. If this were the case, we would have observed decay of the effect as subjects adapted to the context, and the amount and direction of the time-to-peak GF shift would have been the same for 180° and 90° visual feedback rotation. Future work should address the extent to which incongruency in the information provided by two or more sensory modalities, such as the one we studied here, may eventually induce adaptation, leading participants to increase their reliance on the somatosensory-induced prediction of peak LF.

### Visual and Somatosensory Feedback of Object Motion Are Differently Reweighted in Upward and Downward Incongruent Contexts

Our findings suggest that visual and somatosensory feedback are differently integrated for predictive force control as a function of the direction of object displacement, i.e., upward vs. downward motion. Specifically, we observed a weaker effect of visual feedback on GF modulation for UD than DU incongruent trials (Figures 3B, 4, 6B). We propose that reweighting of somatosensory and visual feedback could account for the observed asymmetric effect of visual feedback rotation as a function of movement direction (Sciutti et al., 2012; Toma et al., 2015).

During UD incongruent movements peak LF occurs at the beginning of object motion, due to the initial acceleration peak aimed at overcoming inertia and gravitational pull. In contrast, in DU incongruent movements peak LF occurs towards the end of the movement, i.e., during the deceleration phase. Also, the two contexts are characterized by opposite effects of gravity associated with the actual object motions, i.e., UD and DU performed against and in the same direction of gravity, respectively. Previous work has shown that incongruent visual and somatosensory signals of hand motion are differentially weighted depending on whether movements are facilitated or interfered with by an external force (Di Luca et al., 2011). Furthermore, the consequences of erroneous prediction of the time-to-peak LF are greater in UD than DU trials. This is because in UD incongruent movement prediction errors can result in object slip caused by the asynchrony between peak load and GFs. Conversely, grasp stability would not be challenged to the same extent in DU incongruent movements because GF is already elevated at the time of peak LF. This interpretation is consistent with previous evidence suggesting that, in manipulation contexts where feedback from multiple sensory modalities is available, their integration is sensitive to the cost of making a different kind of errors (Safstrom, 2004).

### Visual Input of Object Motion and Arm-Grip Force Coupling

It might be argued that the observed effects of visual feedback rotation on GF modulation could have resulted from visually-induced changes in arm dynamics. We explored this possibility but found no significant difference in peak shoulder and elbow torques or their relative timing when comparing baseline and incongruent conditions. Thus, the lack of changes in the timing of arm dynamics demonstrates that our results were not caused by changes in the obligatory inertial coupling of arm and GFs across conditions (Danion, 2004, 2007). If this were the case, the observed changes in GF modulation should be entirely explained by similar modulations of arm kinematics, dynamics, and LF during incongruent trials. Following this interpretation, GF would be adjusted accordingly to inertial force changes induced by rotation of visual feedback motion. Predictions of the influence of visual feedback on arm kinematic are based on previous work demonstrating that, during vertical pointing movements, incongruent visual feedback of motion direction elicits direction-dependent modulation of arm kinematics (Sciutti et al., 2012). Our findings of different effects of visual feedback rotation on GFs timing with respect to arm kinematic (i.e., object velocity and movement amplitude) and dynamic (i.e., shoulder/elbow torque) rule out this possibility.

Furthermore, while the lack of influence of 180° visual feedback rotation on arm kinematic (Figure 2B, bottom) seems to contradict previous work (Sciutti et al., 2012), it supports the hypothesis of sensory reweighting processes underlying sensory-motor control (Sober and Sabes, 2003; Sciutti et al., 2012; Toma et al., 2015). Specifically, we speculate that the presence of salient tactile inputs involved in our manipulation task might have increased the weight of non-visual cues. This might explain the finding of reduced influence of vision on arm kinematic, i.e., visual gravity, in the present work with respect to tasks where tactile signals are not involved (Sciutti et al., 2012; Toma et al., 2015).

Therefore, the observation of a weaker influence of vision on arm kinematics during upward movements relative to downward is not surprising. While tactile inputs during upward arm movements inform the system about actual motion dynamic at the very beginning of arm/object motion, during downward movements tactile signals provide salient information about manipulation dynamic only during the deceleration phase, i.e., towards the end of the movement. Thus, our findings of different modulations of arm-grip kinematic and dynamic suggest that visual and tactile-proprioceptive inputs might be interpreted differently according to task demands.

While our results do not fully support the hypothesis of independent control mechanisms for arm dynamic and GFs (Crevecoeur et al., 2016), they suggest that integration of online feedback from multiple sensory modalities, and their relative weight, might be pre-set to task demands. This interpretation is consistent with previous evidence suggesting that, during manipulation, the relevance of a specific source of sensory information on the sensorimotor transformation is regulated to satisfy task requirements (Safstrom, 2004). Our results are also consistent with previous work showing different sensitivity of arm trajectory and GF adjustments in response to self-generated and external dynamic perturbations (Flanagan and Lolley, 2001; Danion, 2007; Crevecoeur et al., 2010, 2016; White, 2015).

Our finding shares some similarities with but also differs from, that reported by Crevecoeur et al. (2016). These authors studied how individuals compensate for the effects of an unpredictable mechanical perturbation delivered to the arm by measuring muscle activity and GFs. Similar to our study, these authors found GF adjustments to be modulated as a function of perturbation direction, rather than employing a default grip correction strategy. However, differently from our study, Crevecoeur and colleagues found that reflex responses of arm and hand were coordinated, as indicated by the near-synchronous temporal coupling between shoulder muscle activation and digit forces. In contrast, we found that the timing of GF, but not the shoulder or elbow torque, was affected by rotated visual feedback. There are four major differences between the study by Crevecoeur et al. (2016) and our study: our subjects: (1) could predict the self-generated LF perturbation induced by vertical object movements; (2) assumed the timing of LF changes by relying on rotated visual feedback—in addition to the veridical somatosensory feedback; (3) experienced a mismatch between visual and somatosensory feedback of hand-held object motion; and (4) our paradigm engaged different circuits supporting visuomotor control, rather than probing reflexes at latencies too short to be influenced by vision. We believe that these methodological differences might underlie the different results in the extent of temporal coupling between arm and hand control. Nevertheless, the results of this previous work and ours can be interpreted using the same conceptual framework of optimal feedback control (Todorov and Jordan, 2002). Specifically, the results of Crevecoeur et al. (2016) and present findings can be accounted for by a control policy that determines how the integration of online feedback from multiple sensory modalities is preset to task demands (Gallivan et al., 2018). In the context of manipulation, it has been demonstrated that the CNS monitors specific sensory events to regulate motor behavior during manipulative actions (Flanagan and Johansson, 2002). Motor reactions to such events depend highly on the context in which the sensory event takes place (Safstrom, 2004). In our task, the real LF “perturbation” was limited to the distal component, while rotated visual feedback induced subjects to expect peak LF to occur before or after the real peak LF. In the study by Crevecoeur et al. (2016), the perturbation to the arm would have mechanically affected both proximal and distal components, and therefore demanded their reflex responses to be temporally coupled to minimize the risk of losing contact with the object.

### Visual Gravity and Inverse Dynamics

Our findings indicate that visual feedback of object motion direction affects predictive GF control. Importantly, we have shown that the change in GF modulation could not be attributed to obligatory coupling of grip and arm forces, nor a change in the time course of shoulder or elbow torques. This implies that the CNS implemented anticipatory GF adjustments by linking visual inputs of object motion to the prediction of force through an inverse-dynamics model of hand-object interaction. Thus, according to this framework, the CNS would select the most appropriate motor commands based on the task kinematic, the associated sensory input, and an internal representation of body kinetic and environmental dynamics (Johansson and Flanagan, 2009; Gallivan et al., 2018). In the context of the present study, visual and somatosensory inputs of the rotated and veridical object kinematics combined with an internal representation of gravity, would enable predictions of upcoming LF changes, and therefore GF adjustments.

Our finding of the influential role of visual input for digit force control is in agreement with previous studies investigating hand-object interactions and haptic perception (Sarlegna et al., 2010; Di Luca et al., 2011; Takamuku and Gomi, 2015; van Polanen et al., 2019). Takamuku and Gomi (2015), for instance, showed that subjects’ perception of a resistive force correlates with the visually-implied force, rather than motor-related errors induced by the delayed cursor. Importantly, these authors found that perception scaled as a function of the time visual feedback was provided during the reaching movements. These findings, together with those reported by another study using a similar delayed visual feedback design (Di Luca et al., 2011), suggest that the CNS during hand-object interaction takes into account visual inputs of motion to interpret and predict mechanical impedance, i.e., the relation between force and motion.

The current study extends these previous observations from time to space domain by demonstrating that visual feedback of object motion direction can modify the internal representation of task dynamics, leading to gripping force modulation. We note that previous studies interpreted the effects of delayed feedback of cursor motion as inducing predictions associated to a new (virtual) mechanical load (or impedance) acting on the hand (Sarlegna et al., 2010; Di Luca et al., 2011; Takamuku and Gomi, 2015). In contrast, we interpret the present effects of rotated feedback of object motion on GF as reflecting “hard-wired” expectation of the effects of gravity, i.e., visual gravity. This might explain why in previous reports, the effects of imaginary inertial force associated with the delayed cursor motion faded with prolonged exposure to the delayed visual feedback (Honda et al., 2012; Takamuku and Gomi, 2015). In contrast, our visually-induced expectation of LF changes consistently elicited GF modulation that did not subside across trials (however, see above section “Visual Gravity Contributes to Anticipatory Grip-Force Control”). Moreover, if visual feedback rotation would drove GF adjustments only due to a new representation of the relationship between force and position, we would expect to observe velocity (inertial) rather than direction (gravity) dependent digit force modulations. Our results rule out this possibility by showing that modulation of GF timing occurred only when visual feedback was vertically rotated.

This interpretation is in agreement with previous behavioral work investigating object manipulation in different conditions of movement acceleration and frequency as well as to object mass, viscosity and gravitational forces, proposing that gravitational and inertial constraints could be separately represented by the CNS (Flanagan and Wing, 1993; Augurelle et al., 2003; Zatsiorsky et al., 2005; Crevecoeur et al., 2009, 2010). Similarly, neurophysiological studies have shown that the CNS can predict the effect of gravity independently from mass and acceleration through a network composed of the visual primary areas, the vestibular nuclei and posterior cerebellum (Angelaki et al., 2004; Indovina et al., 2005; Miller et al., 2008). Recent fMRI studies also demonstrated the role of the insula in controlling vertical vs. horizontal movements (Rousseau et al., 2016) and highlighted different roles of the insula in contexts where actual feedback is available or the subject performs motor imagery (Rousseau et al., 2019). Together this evidence supports our interpretation that the effect of visual feedback rotation on the timing of GF modulation depends on the hard-wired role of an internal representation of gravity underlying predictions of the expected effects of gravitational acceleration.

One of the most accredited hypotheses of how the brain could adjust GF is that the CNS builds an internal representation of load and GF changes based on sensory inputs about inertial and gravitational forces (Augurelle et al., 2003; White et al., 2005; White, 2015). In our case, sensing of the gravitational forces on the object and arm partly occurred through inference based on visual feedback of object motion. We conclude that visual gravity is an important factor driving the inverse transformation of visual object motion into GF control.

## Concluding Remarks

The present study showed that a spatial mismatch between somatosensory and visual cues of object motion direction influenced predictive control of GF modulation. The role of visual feedback for internal representation of task dynamics has been previously investigated (Sarlegna et al., 2010; Honda et al., 2012; Takamuku and Gomi, 2015; van Polanen et al., 2019). However, these studies focused on the temporal congruency between visual and somatosensory signals during manipulation. The novel contribution of the present work is the demonstration that congruency between somatosensory and visual feedback of object motion direction underlies the internal representation of task dynamics. The present findings also extend previous evidence on the role played by visual gravity and multisensory integration (Papaxanthis et al., 1998; Gaveau and Papaxanthis, 2011; Sciutti et al., 2012; Toma et al., 2015) for digit force control involved in object manipulation.

## Data Availability Statement

All datasets generated for this study, supplementary materials and main codes used for the data analysis are available in the OSF repository, https://osf.io/hr2bj/files/.

## Ethics Statement

The studies involving human participants were reviewed and approved by Office of Research Integrity and Assurance at Arizona State University. The patients/participants provided their written informed consent to participate in this study.

## Author Contributions

ST conceived and designed the research. ST set up the experimental apparatus. ST and VC performed the experiments. ST, VC, and MS performed the data analysis. ST and MS interpreted the results. ST and MS prepared the figures. ST and MS drafted the manuscript. All authors edited and approved the final version of the manuscript.

## Conflict of Interest

The authors declare that the research was conducted in the absence of any commercial or financial relationships that could be construed as a potential conflict of interest.
