# Supplementary material for: Visual Feedback of Object Motion Direction Influences the Timing of Grip Force Modulation During Object Manipulation
Source: Front Hum Neurosci. 2020 May 29;14:198. doi: 10.3389/fnhum.2020.00198 (PMC7272672; doi:10.3389/fnhum.2020.00198)
Supplement: Supplementary file 1 [file Data_Sheet_1.PDF]

SUPPLEMENTAL DATA

VISUAL FEEDBACK OF OBJECT MOTION DIRECTION INFLUENCES THE TIMING OF GRIP FORCE MODULATION DURING OBJECT MANIPULATION

Toma S., Caputo V, Santello M.

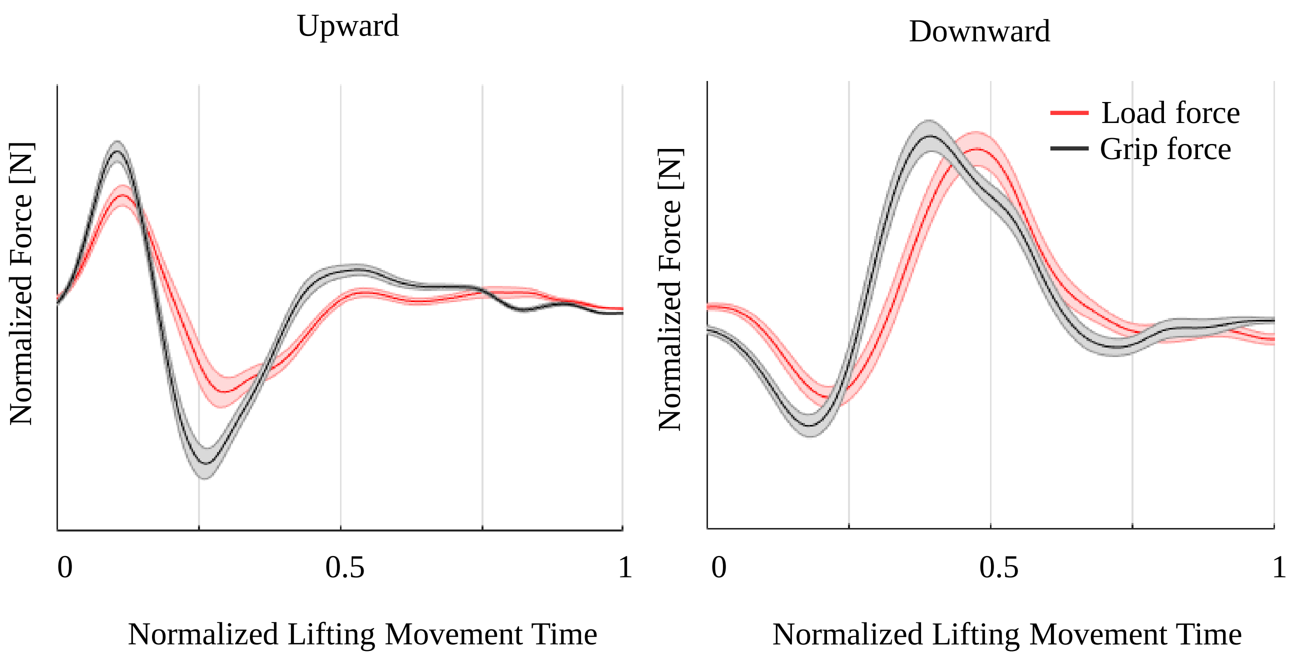

Fig S1. **Digit force modulation during vertical hand-held object movements.** Grip (black) and load (red) force profiles of a representative participant performing upward and downward object motion. As it can be noted digit force profiles are characterized by two distinct temporal pattern of force modulation, i.e., force peaks occurring at the beginning and towards the end of upward and downward motion, respectively. Load force modulation results from the relation among object mass, motion kinematic and gravitational forces. The sensorimotor system is able to predict load force modulation, hence adjusting grip force peaks in anticipation to load force. Line and shaded area of each profile depicts median ±SE of force modulations observed across 25 object motions of the same representative participant. Normalized lifting movement time represent movement onset (0) and movement end (1), i.e., object linear velocity greater and lower than 2% of peak velocity, respectively.

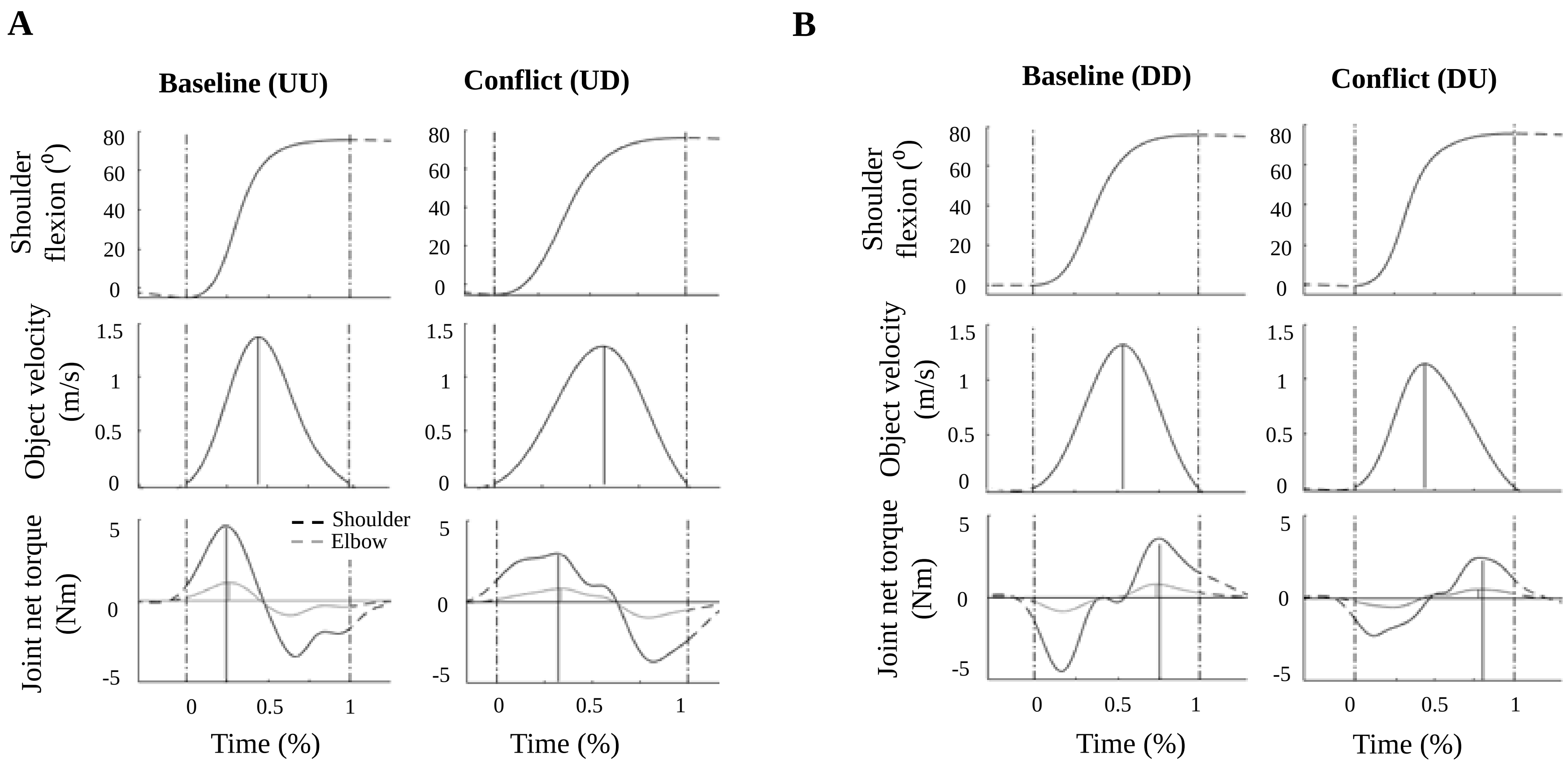

Fig. S2. **Representative kinematic and kinetic data.** From top to bottom, plots show normalized time courses of shoulder flexion, object velocity, and joint net torques from representative trials (1 subject) performing an upward and downward movement (A and B, respectively). In each plot, left and right dashed lines denote onset and end of object movement, respectively. In A and B, the first column shows data from the baseline condition whereas the second column shows data from incongruent visual feedback condition.

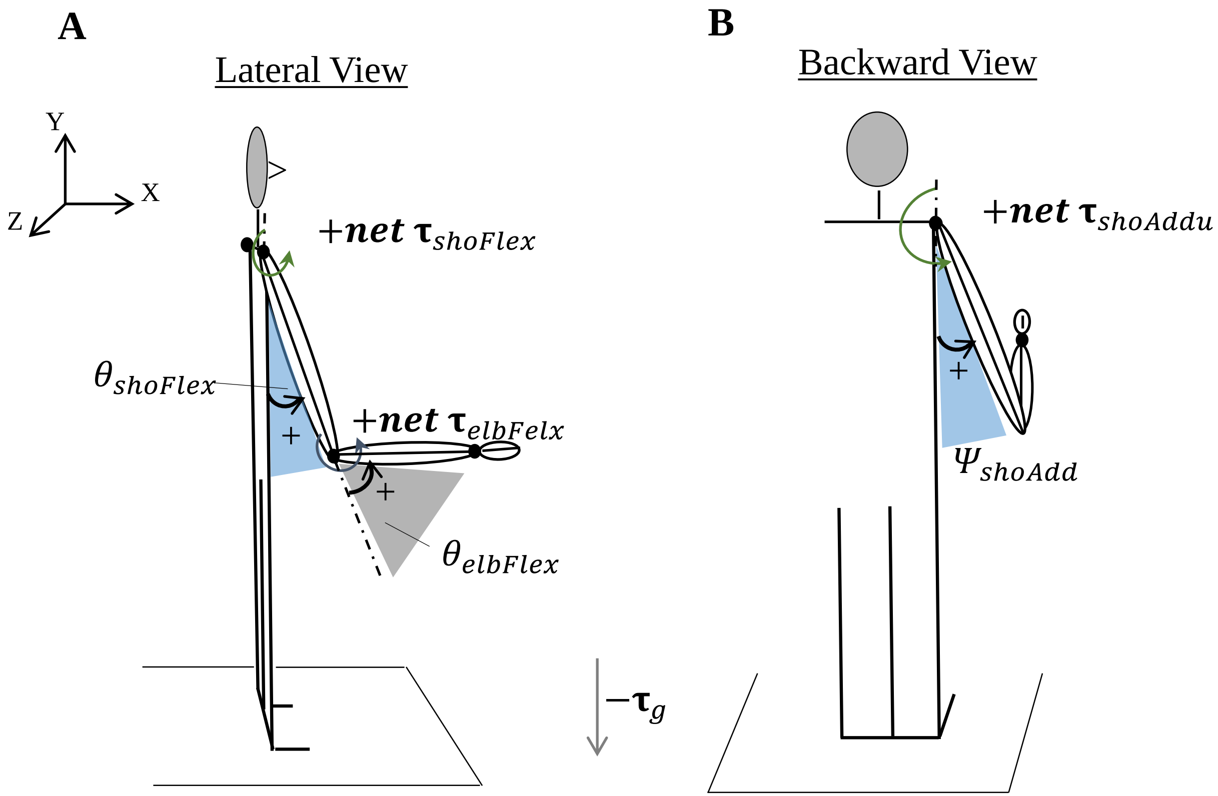

Fig S3. **Definition of joint angles of the arm model.** $\theta_{shoFlex}$ and $\theta_{elbFlex}$ are shoulder and elbow flexion angles, respectively, whereas Ψ_{shoAdd} is the shoulder adduction angle. $\boldsymbol{net}\ \boldsymbol{\tau}_{shoFlex}$ and $\boldsymbol{net}\ \boldsymbol{\tau}_{elbFelx}$ represent the total flexion torque generated by the subject at the shoulder and elbow joint respectively. $\boldsymbol{net}\ \boldsymbol{\tau}_{shoAddu}$ describes the total shoulder joint adduction torque. -$\boldsymbol{\tau}_g$ indicates gravitational forces acting on the arm. For graphical purpose, subjects' arm configuration is different from the straight arm configuration used by the subjects to perform the movement, i.e., rotation around the shoulder joint. **A)** and **B)** depict lateral and backward view, respectively, of the joint angles characterizing the arm model.

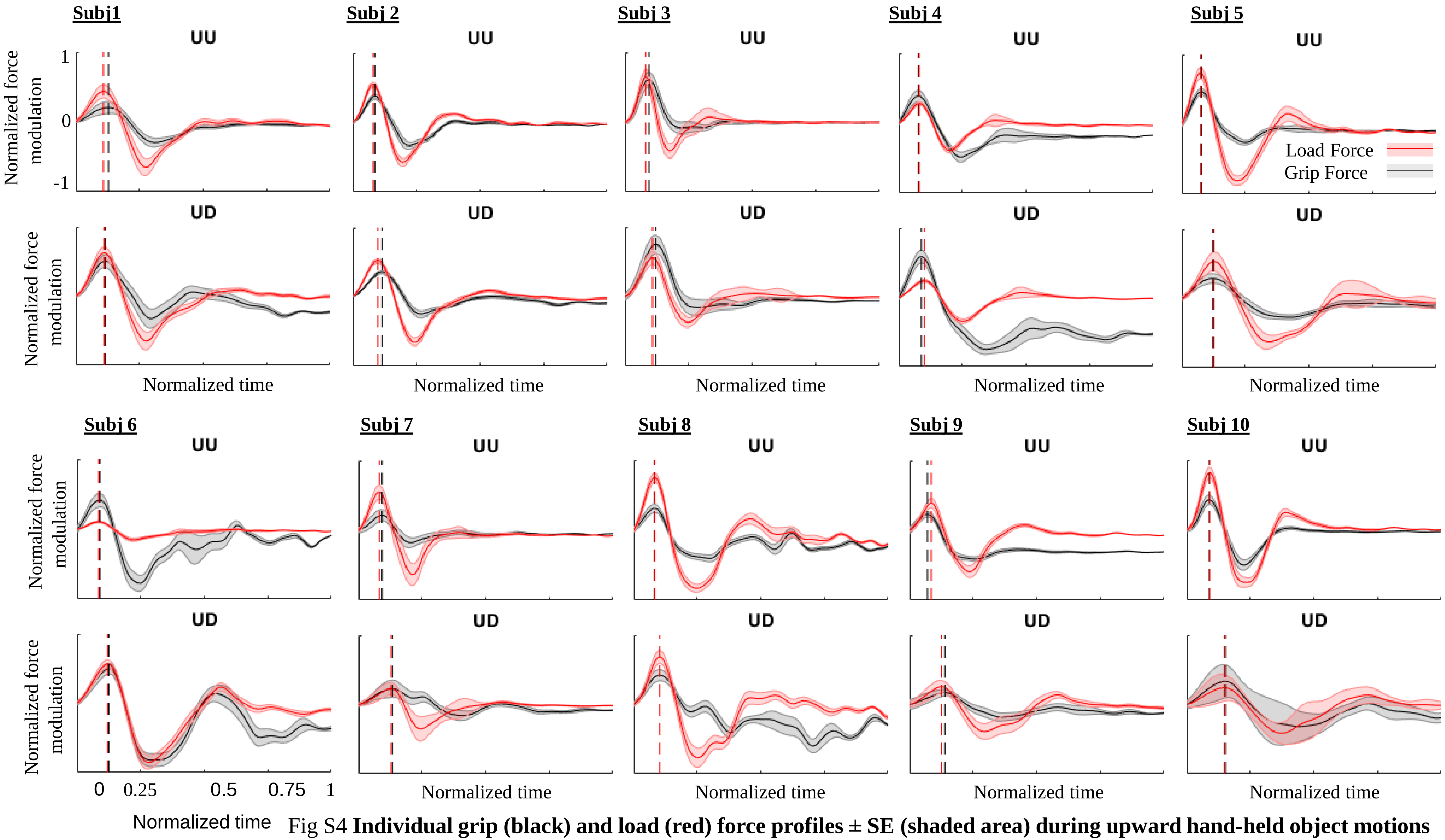

Fig S4 **Individual grip (black) and load (red) force profiles ± SE (shaded area) during upward hand-held object motions**

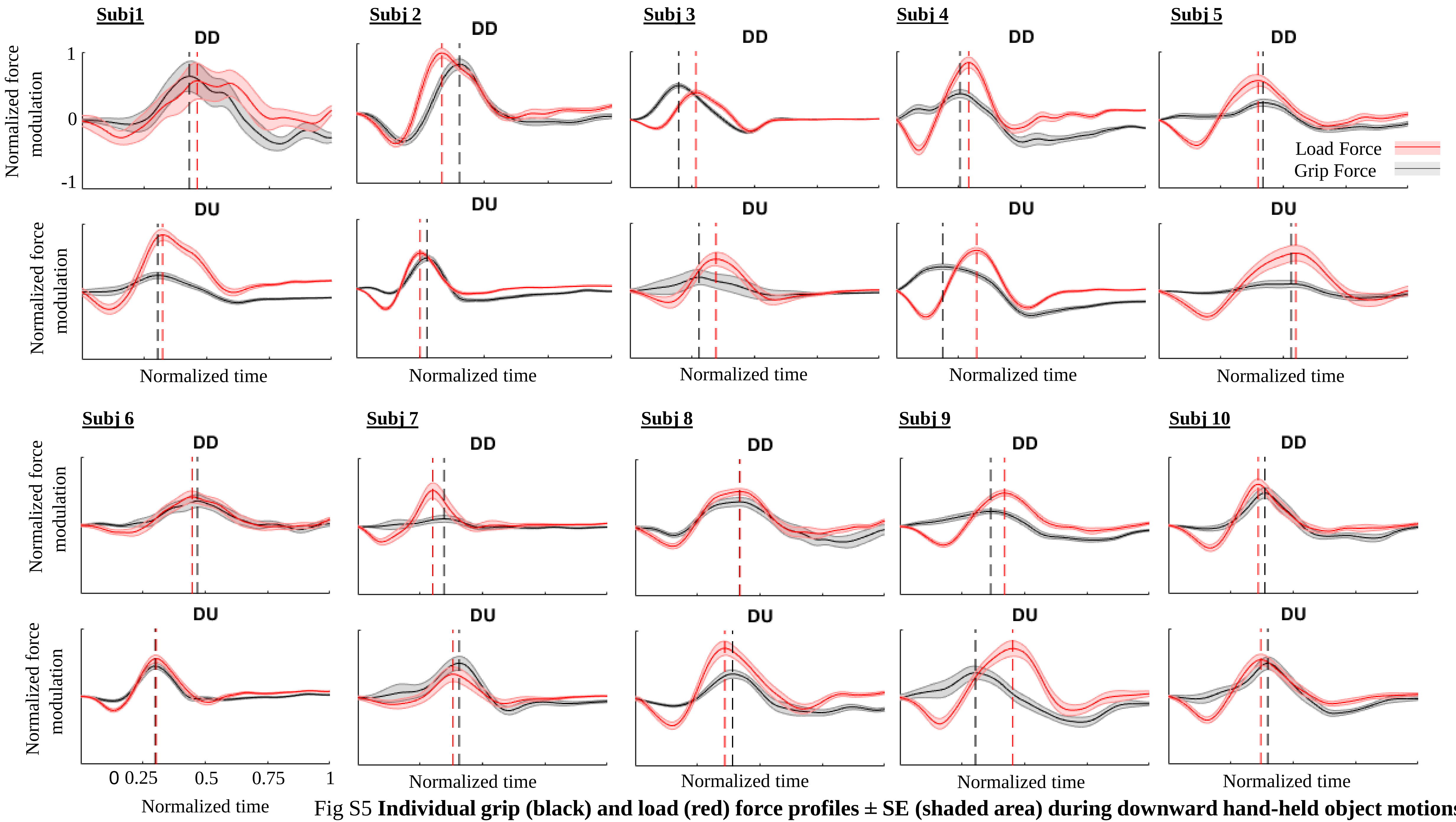

Fig S5 **Individual grip (black) and load (red) force profiles ± SE (shaded area) during downward hand-held object motions**

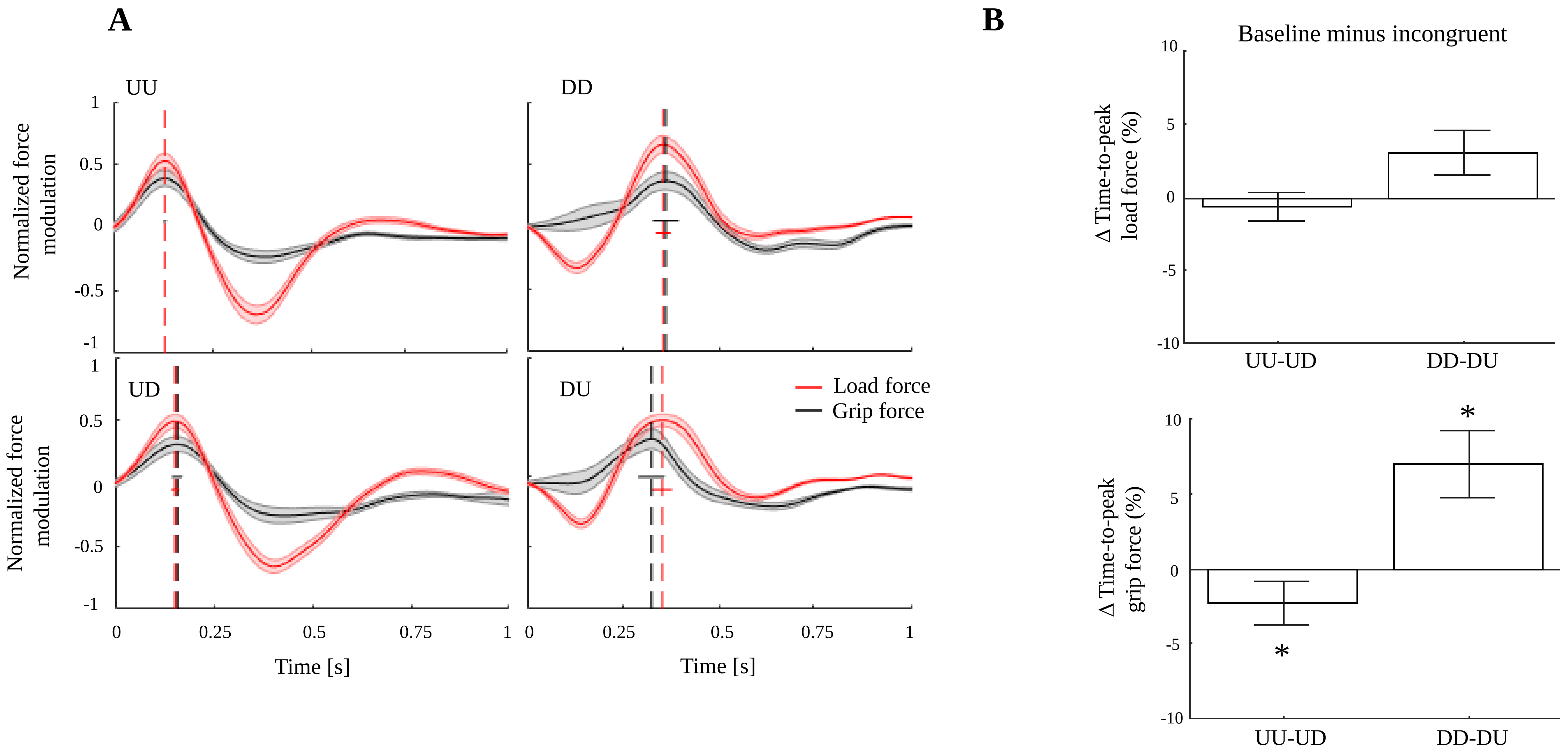

Fig. S6. **Digit force modulation profiles and time-to-peak statistics.** Across subjects (n =14) median ±SE of load (red trace) and grip (black trace) force time course during object displacement (A) and associated statistics (B). Data from those 5 subjects who participated to the control and main experiment (repeated measures participants) were excluded from this analysis. Data are plotted in the same format as Figure 2 and 3 (main text). As it can be noted by comparing these results with those depicted in Figure 3, the group of 14 subjects resemble the overall digit force modulation (A) and time-to-peak force distribution (B) observed in the entire group tested in the main experiment (n = 19). In *A*, black and red vertical lines denote across-subjects median time-to-peak grip and load force, respectively. In *B* Median and CI values of time-to-peak load and grip force were computed as the difference between baseline (congruent) and incongruent trials. Asterisk denotes median values significantly different than zero (Wilcoxon sign-rank test, $p < 0.05$).

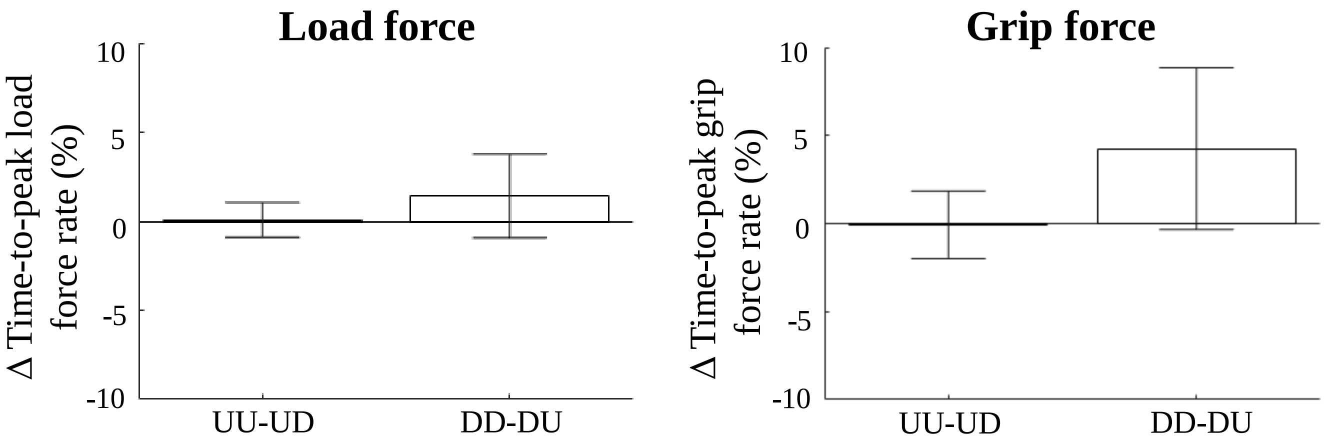

Fig. S7. **Time-to-peak grip and load force rate.** Median and CI values of time-to-peak load and grip force rate (left and right plot, respectively) computed as the difference between baseline and incongruent trials. Data are shown in the same format as Figure 3B, main text.

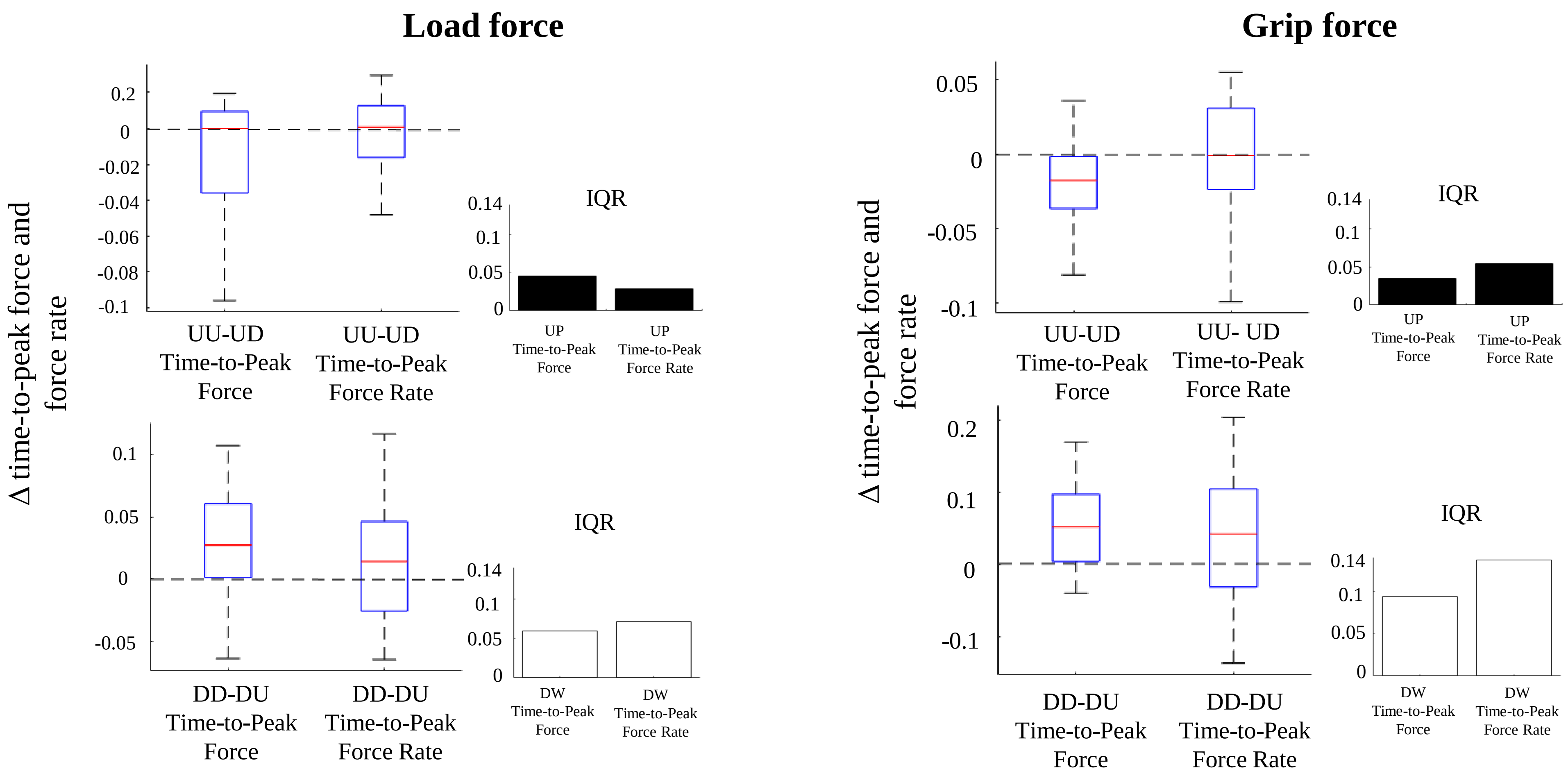

Fig. S8. **Comparison between time-to-peak force and force rate variance.** Across-subjects and -trials variance of the Δ values distribution of time-to-peak force and force rate. In each plot, red horizontal lines denote median values. Upper and lower limits of blue boxes denote the first and third quartile, whereas the whisker were set to cover 1.5 the inter-quantile range (IQR). Note that Δ time-to-peak grip force rate variance is always higher than time-to-peak force. Moreover, time-to-peak grip force rate IQRs are higher than the IQRs associated with load force Δ values.

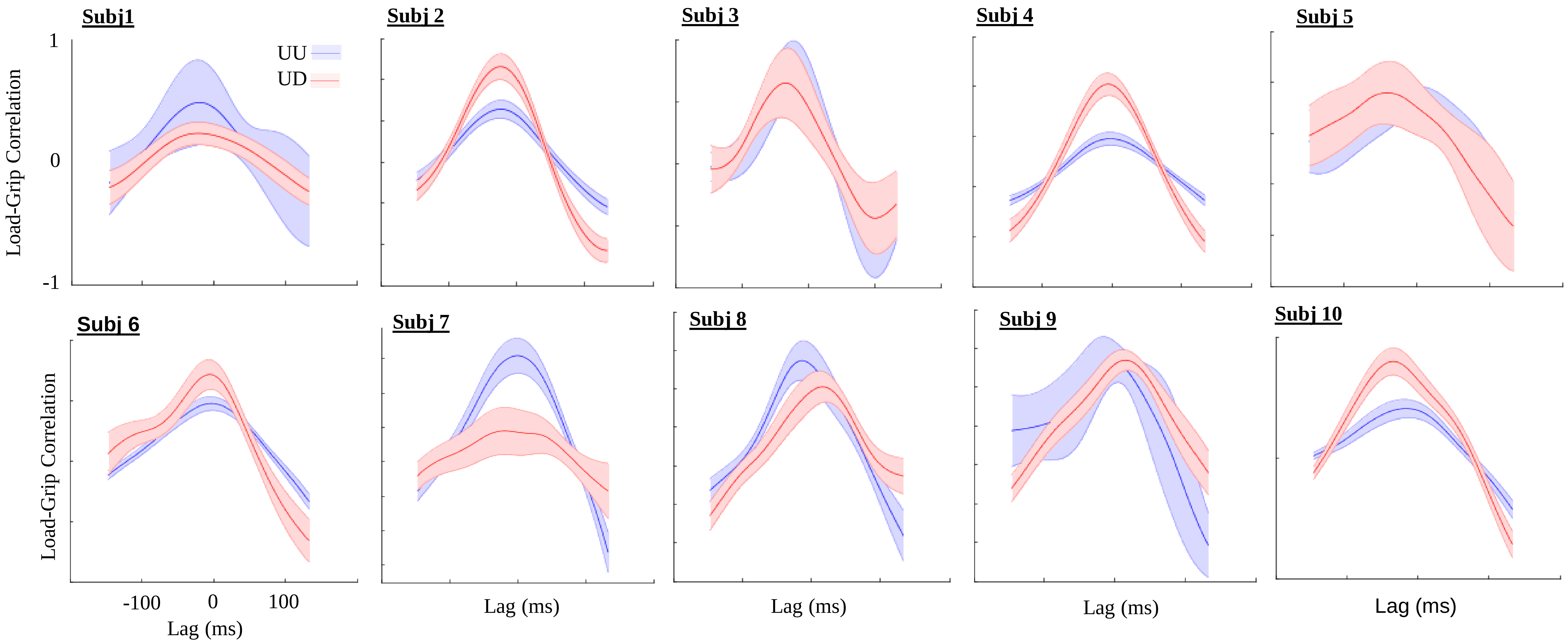

Fig S9 **Individual load-grip force correlation ± SE during baseline and incongruent upward hand-held object motions**

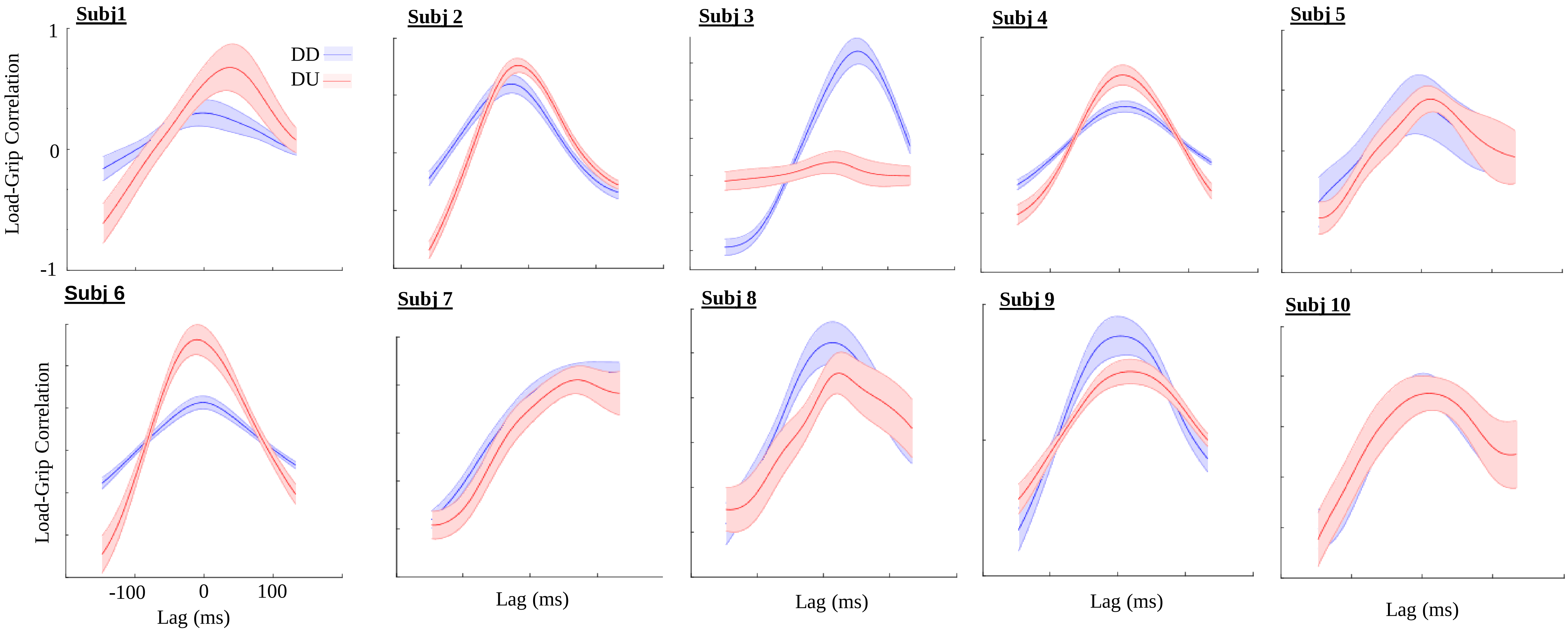

Fig S10 **Individual load-grip force correlation ± SE during baseline and incongruent downward hand-held object motions**

14 subjects

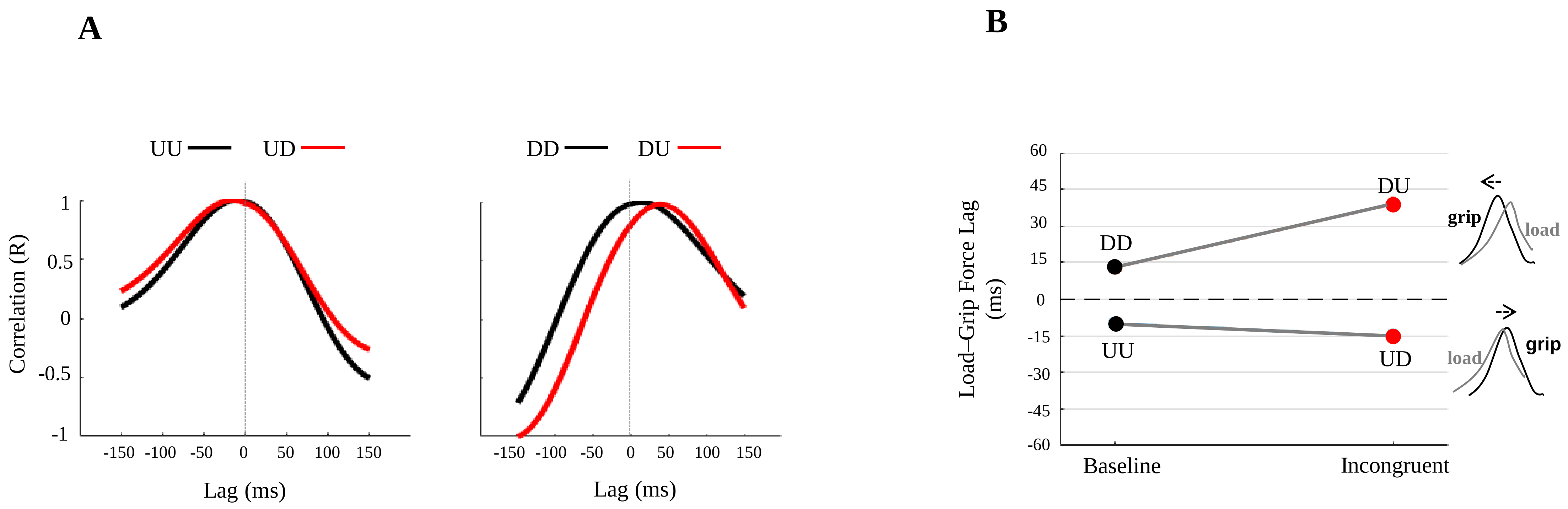

Fig S11. **Cross-correlation analysis: load-grip force time lag across conditions.** Analysis performed on the group of participants that only took part of the main experiment (n = 14)**.** A) Each curve describes lag and correlation between grip and load force profiles obtained during baseline (black) and incongruent (red) conditions. Cross-correlations curves were obtained from across subjects' median force profiles (**Fig. S6**). Positive and negative lag values indicate that GF precedes or follows LF, respectively. B) Opposite effect of 180° visual feedback rotation on Load-Grip lag, i.e., delayed and anticipated grip force modulation with respect to load force during upward and downward object movement, respectively. As it can be noted by comparing these results with those depicted in Figure 4, the exclusion of the 5 repeated-measure participants did change the trend characterizing our main findings, hence excluding a potential contamination of our mixed design.

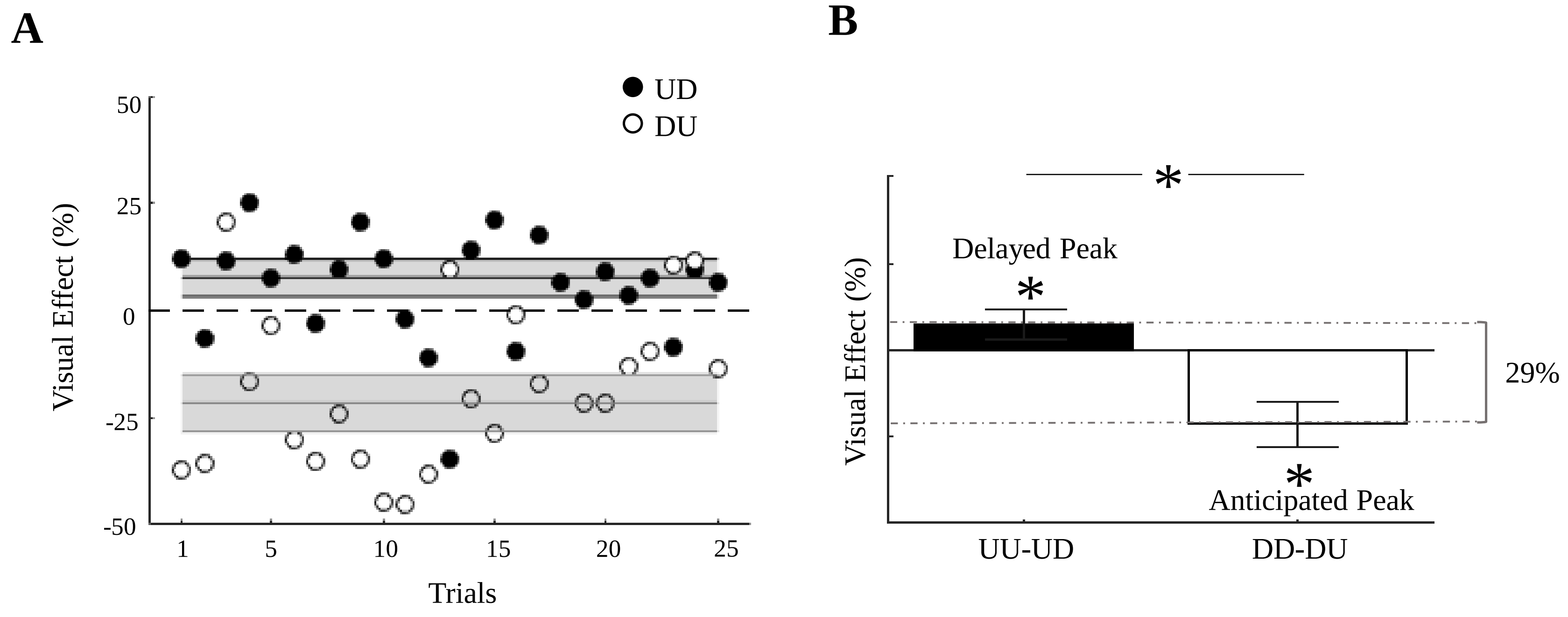

Figure S12. **Visual Effect (VE) of 180° rotation of visual feedback of object motion.** VE across trials modulation (A) and overall statistics (B) exhibited by the group of participants that only took part of the main experiment (n = 14). A) Percentage change of time-to-peak grip force relative to baseline as a function of trial during UD and DU conditions (filled and empty symbols, respectively). Each symbol denotes median value across subjects and trials. B) Median and CI of time-to-peak grip force shift shown in A. Asterisk denotes median values significantly different than zero (Wilcoxon sign-rank test, $p < 0.05$). Asterisk between bars indicates a statistically significant interaction between feedback rotation and movement direction. In A and B, non-zero values denote a shift of the time-to-peak grip force, where positive and negative values denote delay and anticipation relative to baseline, respectively. As it can be noted by comparing these results with those depicted in Figure 6, the exclusion of the 5 repeated-measure participants did not influence the trend characterizing our main findings, hence leading to exclude a potential contamination due to our mixed design.

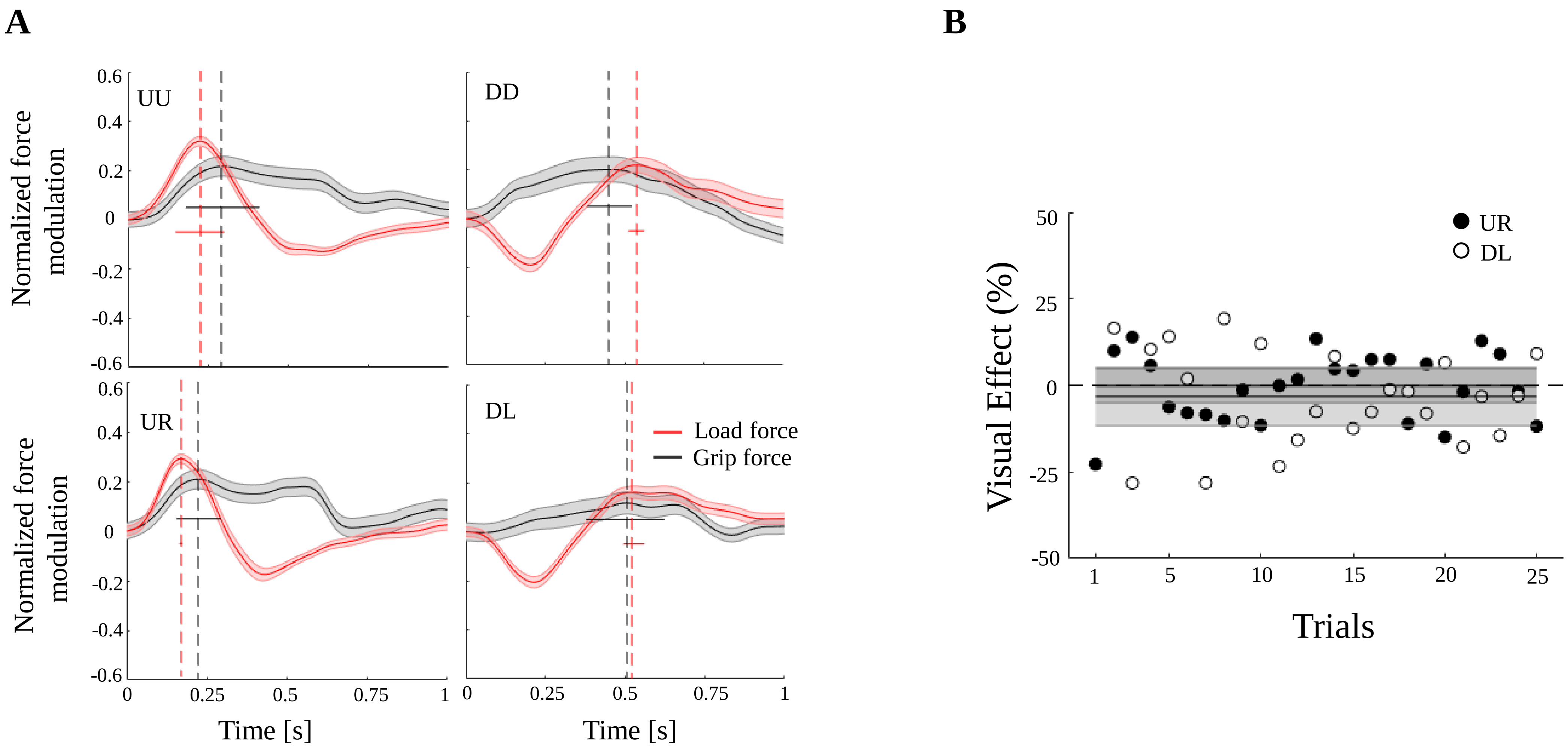

Fig. S13. **Digit force modulation during 90° rotation of visual feedback of object motion.** A) Across-subjects median ±SE of load (red trace) and grip (black trace) force time course during object displacement. For each subject, the static force component was subtracted from each profile (see text for details) and normalized in time and amplitude with respect to their maximum values exhibited in the same condition. Profile onset and end were defined according to the movement onset and end threshold criterion, i.e. 2% of velocity profile peak. Black and red vertical lines represent across-subjects median time-to-peak grip and load force, respectively. B) Percentage change of time-to-peak grip force relative to baseline as a function of trial during UD and DU conditions (filled and empty symbols, respectively). Each symbol denotes median values across subjects and trials.

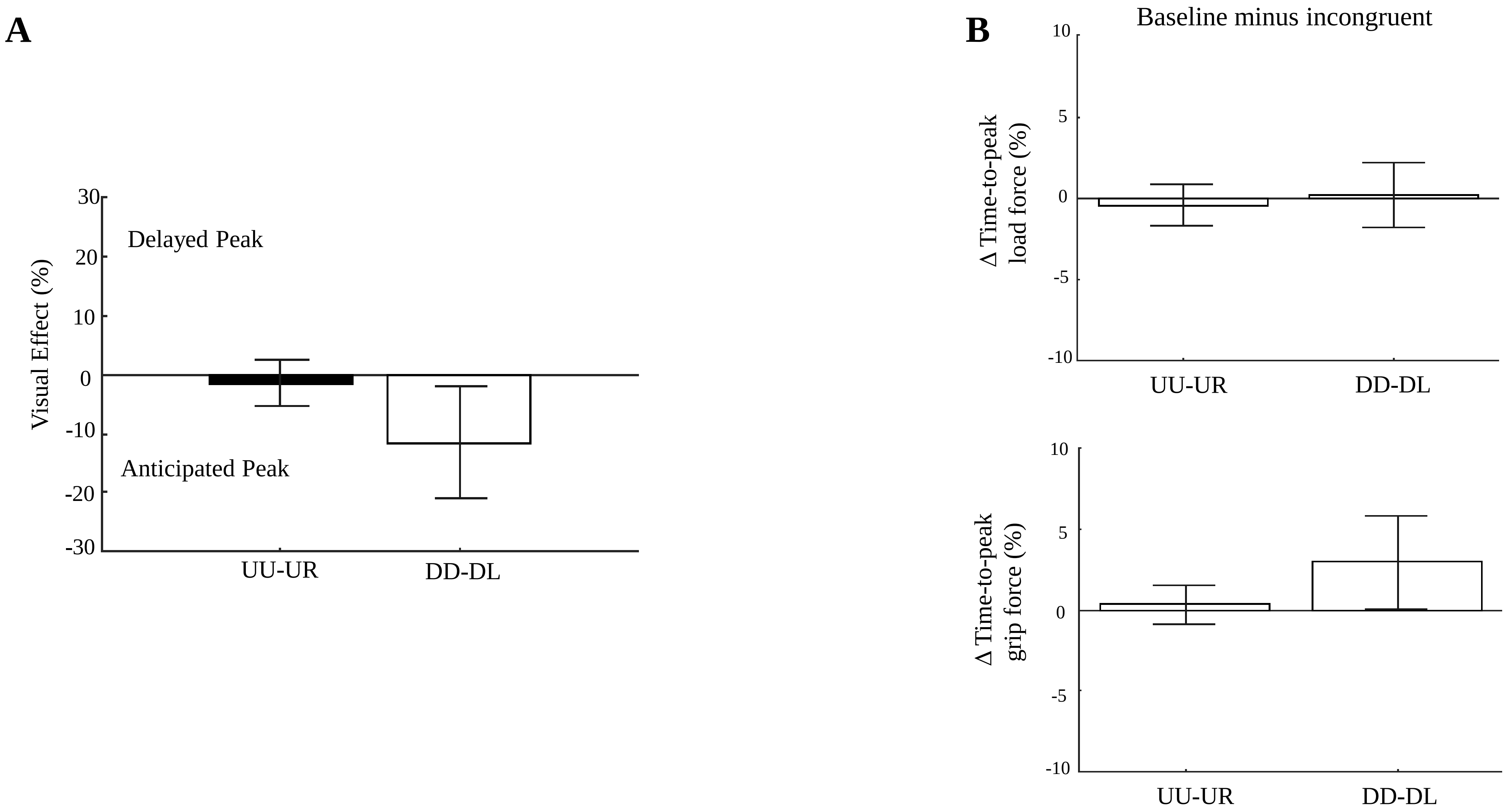

Figure S14. **Influence of 90˚ rotation of visual feedback of object motion on peak force timing.** A) Median ± CI of visual effect on the time-to-peak grip force extracted from the group of participants that only took part on the control experiment (n = 14). B) Median and CI values of time-to-peak load and grip force (top and bottom plot, respectively) computed from the same participants sample. As it can be noted by comparing these results with those depicted in Figure 7, the exclusion of the 5 repeated-measure participants did not influence the trend characterizing our main findings, hence leading to exclude a potential contamination due to our mixed design.

**Link**	**DOF**	**αi**	**ai**	**θi**	**di**	**Type**	**Off-set**
1	Sh X	π/2	0	π/2	0	P	0
2	Sh Y	π/2	0	π/2	0	P	0
3	Sh Z	π/2	0	π/2	0	P	0
4	Sh adduction	π/2	0	0	0	R	- π/2
5	Sh flexion	π/2	0	0	0	R	π/2
6	Sh external rotation	π/2	0	0	Lu	R	π
7	El flexion	0	Lf	0	0	R	π/2

Table S1. **D-H parameters for each link of the model.** *Sh:* shoulder; *El:* elbow; *Lf:* forearm length; *Lu:* upper arm length; *P:* prismatic joint; *R:* revolute joints. *a:* link length, α: link twist, *d:* link offset and *θ:* link joint angle.

Subjects	**1**	**2**	**3**	**4**	**5**	**6**	**7**	**8**	**9**	**10**	**11**	**12**
Height (cm)	160	165	175	175	178	170	180	165	175	183	173	188
Weight (kg)	68	74	68	59	82	68	68	91	68	58.5	86	91
Lu (cm)	32	29	31	30	31	32	30	32	29	32.3	35	28
Lf (cm)	25	23	23	26	26	25	23	25	25	27	22.5	27
Lh (cm)	18	19.5	18	19	20	18	18	18	20.5	19.5	19	21
Mu (kg)	1.52	2.1	1.57	1.54	2.5	2.02	1.78	1.93	1.78	1.3	2.69	2.15
Mf (kg)	0.82	0.9	0.73	1.01	1.3	0.97	0.69	0.83	1.06	0.9	0.88	1.37
Mfh+obj (kg)	1.55	1.76	1.5	1.86	2.2	1.81	1.49	1.55	1.98	1.78	2.42	2.33
rU (cm)	13.5	12.2	13.1	12.66	13.02	13.5	12.66	13.5	12.23	13.6	14.7	11.81
rF (cm)	19.2	19.3	19.28	20.71	20.45	20.04	20.23	19.2	21.29	22.7	25.6	21.42
l(lo) U (kg cm^2 s^{-2})	35.1	39.2	35.7	29.7	45.12	35.5	35.9	50.5	35.7	29.78	47.5	51.52
l(ap) U (kg cm^2 s^{-2})	97.3	114.2	119.9	105.9	146.35	112.42	127.5	140.7	119.98	117.25	145.03	175.5
l(tr) U (kg cm^2 s^{-2})	86.6	102.4	106.7	93	132.1	100.01	113.44	128.4	106.7	102.98	131.48	159.25
l(lo) F (kg cm^2 s^{-2})	16.86	18.8	16.06	12.6	21.2	16.32	15.8	25.38	16.06	12.01	23.04	24.16
l(ap) F (kg cm^2 s^{-2})	213.01	212.8	195.44	226.1	306.46	246.75	184.17	237.04	232.6	236.24	290.77	294.8
l(tr) F (kg cm^2 s^{-2})	221.2	222.1	203.45	232.4	316.9	254.83	192.12	249.25	240.6	242.47	301.9	306.77
Experiment type	180˚	90˚	180˚	90˚	90˚	180˚	180˚	180˚	90˚	180˚	180˚	90˚

Table S2. **Arm model parameters from 12 subjects.** *Lu*: upper arm length; *Lf*: forearm length; *Lh*: open hand length; *Mu*: upper arm mass; *Mf:* forearm mass; *Mfh+obj:* sum of forearm, hand and object mass; *rU*: upper arm center of mass (link-6); *rF:* forearm center of mass (link-7); *I(lo) U:* inertia along the longitudinal axis of the upper arm*; I(ap) U:* inertia along the antero-posterior axis of the upper arm; *I(tr) U:* inertia along the transversal axis of the upper arm;); *I(lo) F:* inertia along the longitudinal axis of the forearm*; I(ap) F:* inertia along the antero-posterior axis of the forearm; *I(tr) U:* inertia along the transversal axis of the forearm; *Experiment type: 180˚ or 90˚ visual feedback rotation*
